# Metabolic engineering of *Clostridium ljungdahlii* for the production of hexanol and butanol from CO_2_ and H_2_

**DOI:** 10.1186/s12934-022-01802-8

**Published:** 2022-05-14

**Authors:** Ira Lauer, Gabriele Philipps, Stefan Jennewein

**Affiliations:** Department for Industrial Biotechnology, Fraunhofer Institute for Molecular Biology and Applied Ecology IME, Forckenbeckstr. 6, 52074 Aachen, Germany

**Keywords:** Syngas fermentation, Biofuels, Wood-Ljungdahl pathway, Butanol, Hexanol, Acetogens

## Abstract

**Background:**

The replacement of fossil fuels and petrochemicals with sustainable alternatives is necessary to mitigate the effects of climate change and also to counteract diminishing fossil resources. Acetogenic microorganisms such as *Clostridium* spp. are promising sources of fuels and basic chemical precursors because they efficiently utilize CO and CO_2_ as carbon source. However the conversion into high titers of butanol and hexanol is challenging.

**Results:**

Using a metabolic engineering approach we transferred a 17.9-kb gene cluster via conjugation, containing 13 genes from *C. kluyveri* and *C. acetobutylicum* for butanol and hexanol biosynthesis, into *C. ljungdahlii*. Plasmid-based expression resulted in 1075 mg L^−1^ butanol and 133 mg L^−1^ hexanol from fructose in complex medium, and 174 mg L^−1^ butanol and 15 mg L^−1^ hexanol from gaseous substrate (20% CO_2_ and 80% H_2_) in minimal medium. Product formation was increased by the genomic integration of the heterologous gene cluster. We confirmed the expression of all 13 enzymes by targeted proteomics and identified potential rate-limiting steps. Then, we removed the first-round selection marker using CRISPR/Cas9 and integrated an additional 7.8 kb gene cluster comprising 6 genes from *C. carboxidivorans.* This led to a significant increase in the hexanol titer (251 mg L^−1^) at the expense of butanol (158 mg L^−1^), when grown on CO_2_ and H_2_ in serum bottles. Fermentation of this strain at 2-L scale produced 109 mg L^−1^ butanol and 393 mg L^−1^ hexanol.

**Conclusions:**

We thus confirmed the function of the butanol/hexanol biosynthesis genes and achieved hexanol biosynthesis in the syngas-fermenting species *C. ljungdahlii* for the first time, reaching the levels produced naturally by *C. carboxidivorans*. The genomic integration strain produced hexanol without selection and is therefore suitable for continuous fermentation processes.

**Supplementary Information:**

The online version contains supplementary material available at 10.1186/s12934-022-01802-8.

## Background

Medium chain alcohols such as butanol and hexanol have the potential to serve as biofuels [1]. Butanol has properties similar to gasoline [2] and is already used as a drop-in fuel [3]. With a higher cetane number and energy density than butanol, hexanol is also considered to be an attractive fuel and can be blended with diesel and used in existing diesel engines [4]. Furthermore, butanol and hexanol can be oligomerized with acetone to form high molecular weight products. These longer-chain ketones (C11–C15) such as 6-undecanone can be used as well in diesel engines or, after hydrotreatment as jet fuel [5]. With about 359 billion liters of fuel consumed and 905 million tonnes of carbon dioxide emitted in 2018, the commercial aircraft sector accounts for ~ 2% of global CO_2_ emissions [7]. Hence, biologically produced butanol and hexanol could contribute to build a carbon–neutral transportation sector. Furthermore, hexanol finds its use in multiple applications, *e.g.* as solvent, plasticizer, pesticide, flavoring ingredient and as building block for chemical synthesis [8, 9].

Today, butanol and hexanol are derived from petrochemical sources, but they can be produced by microorganisms, including those that use CO and/or CO_2_ in combination with H_2_ as a substrate. Acetone, butanol and ethanol have been produced on an industrial scale from substrates such as sugars and molasses by *Clostridium acetobutylicum* for more than 100 years, in a process known as ABE fermentation [10]. Butanol concentrations of 11.7 g L^−1^ and 12.6 g L^−1^ can be achieved in batch fermentation in glucose-based medium using wild-type strains of *C.* *acetobutylicum* [11] and *C.* *beijerincki* [12], respectively. Metabolic engineering has improved the product yields of such microbes. For example, an engineered strain of *C.* *acetobutylicum* produced 17.6 g L^−1^ butanol in fed-batch fermentation with glucose [11], whereas a mutant strain of *C.* *beijerinckii* produced a total of 151.7 g of butanol from 500 g glucose in a 1 L fed-batch process with continuous gas-stripping for product recovery [13]. Metabolic engineering has also been used for alcohol production in species that lack this ability in the wild. For example, the heterologous production of 30 g L^−1^ butanol by *Escherichia coli* was achieved using an optimized glucose fed-batch process with gas-stripping for product recovery [14]. Another *E. coli* strain achieved the heterologous production of 0.047 g L^−1^ hexanol and 6.5 g L^−1^ butanol when provided with glucose in a small scale fed-batch fermentation [15]. Recently, chain elongation to caproate and hexanol in *C.* *saccharoperbutylacetonicum* (a native producer of butyrate and butanol) was enabled by the introduction of *C.* *carboxidivorans* genes, leading to the production of 0.53 g L^−1^ hexanol in the presence of 40 g L^−1^ glucose in a batch fermentation [16].

Although the production of butanol and hexanol from sugars is promising, these substrates are derived from the food and feed industry thus introducing competition for land use and other agricultural resources. An alternative substrate is synthesis gas (syngas), a mixture of CO, CO_2_ and H_2_ often produced as a process gas in the steel industry and by the gasification of organic matters such as municipal waste [17]. Syngas is metabolized by microbes such as *C. ljungdahlii, C. autoethanogenum, C. carboxidivorans* and *Acetobacterium woodii* [18] via the Wood-Ljungdahl pathway to produce the intermediate acetyl-CoA [19, 20]. This central metabolite is subsequently converted to the main fermentation products ethanol and acetate [21] and biomass. Some *Clostridium* strains, such as *C.* *carboxidivorans*, naturally convert acetyl-CoA to hexanol [22], whereas *C.* *kluyveri* produces only traces of hexanol but large amounts of the corresponding acid, caproate [23, 24]. However, *C. kluyveri* is not able to grow on gaseous substrates. The highest titer of hexanol reported on a gaseous substrate thus far is 1.36 g L^−1^ without product extraction [25] and 2.4 g L^−1^ when the product is removed by in situ extraction [26]. In both cases, this was achieved using wild-type *C.* *carboxidivorans*.

The *C. carboxidivorans* operon proposed to enable the conversion of acetyl-CoA to butyryl-CoA/butanol was identified, along with a second homologous operon [27] that we assumed to be responsible for hexanoyl-CoA/hexanol production. Metabolic engineering in *C. carboxidivorans* was only recently reported [28]. The heterologous production of butanol by *C.* *ljungdahlii* [29] and *C.* *autoethanogenum* [30] has been achieved following the introduction of genes from *C.* *acetobutylicum*, resulting in butanol titers of 0.15 g L^−1^ and 1.54–1.90 g L^−1^, respectively. Furthermore, the co-cultivation of *C.* *autoethanogenum* and *C.* *kluyveri* on syngas supplemented with acetate produced 0.26 g L^−1^ d^−1^ butanol and 0.20 g L^−1^ d^−1^ hexanol in a batch fermentation [1]. These rates could even be increased to about 0.73 g L^−1^ d^−1^ butanol and 0.54 g L^−1^ d^−1^ hexanol in a study considering different pH profiles using *C. ljungdahlii* and *C. kluyveri* in continuous fermentation mode [31]. However, the metabolic engineering of *Clostridium* species to produce hexanol on a gaseous substrate has not been reported thus far.

Here we engineered *C. ljungdahlii* for the production of butanol and hexanol from a gaseous substrate. *C. ljungdahlii* is a genetically accessible strain that can use CO_2_ + H_2_ or CO or mixtures of both as a substrate but does not naturally produce medium chain alcohols [29]. Accordingly, we expressed *C.* *kluyveri* enzymes in *C. ljungdahlii* that convert acetyl-CoA (the main product of the native Wood-Ljungdahl pathway in *C. ljungdahlii*) into butyryl-CoA and hexanoyl-CoA, as well as a *C.* *acetobutylicum* bifunctional alcohol dehydrogenase to form the corresponding alcohols. We introduced the genes by conjugation and subsequent genomic integration, and compared the performance of the plasmid-carrying and genomic integration producer strains on gaseous substrates in terms of growth, product formation and protein expression levels. We used targeted proteomics to identify potential bottle-necks in the heterologous pathway. For further genomic integration of additional genes the removal of the introduced selection marker was required, which was accomplished by using the CRISPR/Cas9 system [32]. This was followed by the conjugation and subsequent genomic integration of a second gene cluster from *C.* *carboxidivorans* comprising six genes assigned for butyryl-CoA synthesis from acetyl-CoA [27]. When normalized to the biomass, the final recombinant *C. ljungdahlii* strain was able to synthesize hexanol in comparable amounts to wild-type *C. carboxidivorans* (which naturally produces hexanol from syngas components), without the need for continuous selection.

## Methods

### Culture conditions

*Clostridium *wild-type strains were obtained from the DSMZ (Braunschweig, Germany) and are listed along with the engineered strains in Table 1. *C. kluyveri* DSM 555 was grown in DSM medium 52 in 250-mL serum bottles at 37 °C. *C. ljungdahlii* DSM 13528 was cultivated in modified ATCC 1754 medium without Na_2_S and with 20 mM Bis–Tris buffer instead of sodium bicarbonate. Fructose (5 g L^−1^) or gaseous substrate (20% CO_2_ and 80% H_2_, 1 bar overpressure) were provided as the carbon and energy source. When necessary, 200 mg L^−1^ d-cycloserine, 4 mg L^−1^ clarithromycin and/or 4 mg L^−1^ thiamphenicol were added for selection. During strain engineering, *C.* *ljungdahlii* was cultivated in complex YTF medium (10 g L^−1^ yeast extract, 16 g L^−1^ tryptone, 5 g L^−1^ fructose, 0.75 g L^−1^ cysteine, 4 g L^−1^ NaCl, pH 6.0) [33] at 37 °C in an anaerobic workbench Bactron 600 (Shel Lab, Cornelius, OR, USA) or at 34 °C in a Whitley A35 anaerobic chamber (Don Whitley Scientific, Bingley, UK) with an anaerobic atmosphere consisting of 10% CO_2_, 5% H_2_ and 85% N_2_. Growth experiments with *C. ljungdahlii* were performed in 250-mL serum bottles filled with 25 mL of the appropriate medium. A high gas-to-liquid ratio was chosen in order to promote a higher gas-to-liquid mass transfer and enough gaseous substrate for growth. The inoculated ATCC 1754 medium without fructose was supplied with pre-mixed gas composed of 20% CO_2_ (purity ≥ 99.9%) and 80% H_2_ (purity ≥ 99.999%) (Westfalen, Münster, Germany) with 1 bar overpressure and were incubated at 37 °C on a rotary shaker at 150 rpm. The gas phase was renewed when the pressure in the bottles decreased perceivably. Further information on microorganisms and medium compositions are given in Additional file 1: S1.1. Fermentations were carried out in 3.7-L KLF reactors (Bioengineering, Wald, Switzerland) filled with 2 L modified ATCC 1754 medium at 37 °C, stirred at 300 rpm with a stepwise increase to 500 rpm during the fermentation. A 100 mL volume of culture adapted to grow on gaseous substrate, was used as the inoculum. A constant pH 6.0 (± 0.1) was maintained with 1 M NaOH. Fermentations of the double genomic integration strain were supplied with 100 mM Bis–Tris buffer. The reactors were continuously supplied with a synthetic mixture of 20% CO_2_ (purity ≥ 99.995%) and 80% H_2_ (purity ≥ 99.999%) (Westfalen) mixed using a Gmix (HiTech Zang, Herzogenrath, Germany) at a flow rate of 0.17–0.25 L min^−1^. The exhaust air was cooled to below 10 °C.

**Table 1:** Strains and plasmids used in this study

	Important features	Source
Strain		
*C. kluyveri* DSM 555	Wild-type	DSMZ
*C. ljungdahlii* DSM 13528	Wild-type	DSMZ
*C. lju* pIM Hex#15	Wild-type *C. ljungdahlii* carrying the pIM Hex#15 plasmid	This work
*C. lju* Hex#15^gInt^	*C. ljungdahlii* genomic integration strain with the Hex#15 gene cluster cassette and *ermC*	This work
*C. lju* Hex#15^gInt^ Δ*ermC*	*C. ljungdahlii* genomic integration strain with the Hex#15 gene cluster cassette, and the *ermC* gene removed using CRISPR/Cas9	This work
*C. lju* Hex#15^gInt^ Ccar1^gInt^	*C. ljungdahlii* double genomic integration strain with the Hex#15 and Ccar1 gene cluster cassettes, and the *ermC* gene removed using CRISPR/Cas9	This work
Plasmid		
pIM Hex#15	Butyryl-CoA/hexanoyl-CoA cluster from *C. kluyveri*, *adhE* from *C. acetobutylicum* (SI 1.3), plasmid backbone with *oriT/traJ*, *catP*, *ermC*, *himar1,* and *repH* (SI 1.2): 25.6 kb	This work
pCJRK Δ*ermC*	*cas9*, gRNA with N20 sequence for *ermC*, homology arms, *catP,* and *repH*: 12.6 kb (SI 1.5)	This work
pIM Ccar1	Butyryl-CoA/hexanoyl-CoA cluster 1 from *C.* *carboxidivorans* (SI 1.4), plasmid backbone with *oriT/traJ*, *catP*, *ermC*, *himar1*, and *repH* (SI 1.2): 15.5 kb	This work

### Cloning of butanol and hexanol pathway genes

To prepare vector pIM Hex#15, *C. kluyveri*, *C. acetobutylicum* and *C.* *ljungdahlii* genes and promoters were amplified from genomic DNA by PCR using appropriate primers and were joined by overlap-extension PCR before insertion into pDONR vectors and transfer to the in-house *Clostridium* destination vector pSLIC-Dest using multisite Gateway technology (Thermo Fisher Scientific, Waltham, MA, USA). The Gram-positive origin of replication (*repL*) was replaced with *repH* to facilitate successful conjugation. Details on cloning of pIM Hex#15 and its precursor plasmids are provided in Additional file 1: S1.2 and S1.3 comprising primer lists (Table S1, Table S3), list of used nucleic acid sequences for hexanol gene cluster Hex#15 (Table S2), overview of the cloning strategy and vector maps of the different plasmids (Fig. S1), and structure of the Hex#15 gene cluster (Fig. S2).

To prepare vector pIM Ccar1, the operon annotated for butanol formation in *C.* *carboxidivorans* [27] and the *thlA1* (CA_C2873) promoter region from *C.* *acetobutylicum* were amplified from genomic DNA using primers listed in Table S 4 in Additional file 1: S1.4 and were joined to the backbone derived from the previously described pIM Hex#15 plasmid using Gibson Assembly [34].

### Conjugation and genomic integration

The vectors were introduced into wild-type *C.* *ljungdahlii* by conjugation with an *E.* *coli* donor as previously described [35] with minor changes (see details in Additional file 1: S1.6). Genomic integration was achieved as previously described [35] with minor changes: The cultures were incubated at 34 °C until cell growth was visible, before incubating at room temperature or 28 °C to favor Himar1 transposase activity [36]. Fresh medium was inoculated with the culture (1:100) every 5–8 days. Genomic DNA of cultures with Hex#15 or Ccar1 construct was isolated after twelve or six consecutive inoculation steps, respectively. For the genomic DNA isolation the NucleoSpin Tissue Kit (Macherey–Nagel, Düren, Germany) was used according to the manufacturer’s instructions.

### Inverse PCR

Inverse PCR was carried out as previously described [35] following the digestion of genomic DNA with *AseI* (New England Biolabs, Ipswich, MA, USA) and re-ligation with 1 U µL^−1^ T4 DNA ligase (Fisher Scientific, Waltham, MA, USA). The ligated DNA was diluted 1:20 and used as the template for amplification with Herculase II DNA polymerase (Agilent Technologies, Santa Clara, CA, USA) using outward-facing primers iPCR_mlsR_for (5′-AGC ACG AGC TCT GAT AAA TAT GAA C-3′) and iPCR_mlsR_rev (5′-ACA TGC AGG AAT TGA CGA TTT AAA C-3′) binding at the edge of the *ermC* gene. PCR products were separated by 1% agarose gel electrophoresis and DNA was extracted from excised bands using the NucleoSpin Gel and PCR Cleanup Kit (Macherey–Nagel, Düren, Germany) before Sanger sequencing at Seqlab (Göttingen, Germany).

### CRISPR/Cas9

CRISPR/Cas9 was used to excise the selectable marker *ermC* from the first genomic integration strain. Therefore, an *E. coli*—*Clostridium* shuttle plasmid was designed providing the necessary components on a single plasmid adapted from information provided by Huang et al*.* [37]. In detail, the plasmid contained *repH* as the origin of replication, *catP* for thiamphenicol selection, *cas9* under the control of a lactose-inducible promoter [38] and the guide RNA (gRNA) with the N20 sequence targeting *ermC*. The N20 sequence of the gRNA was 5′-ATAAGTGAGCTATTCACTTT-3′ and was designed using CRISPRdirect [39]. The plasmid also contained homology arms to promote the repair of the Cas9-induced double-strand break by homologous recombination. For the excision of the 1236-bp *ermC* gene cassette, 623 bp upstream and 791 bp downstream flanking sequences were selected. Primers used for generation of the CRISPR/Cas9 plasmid pCJRK Δ*ermC* can be found in Table S5 in Additional file 1: S1.5.

The plasmid was introduced into *C.* *lju* Hex#15^gInt^ by electroporation as described by Leang et al*.* [33] and positive transformants were selected based on thiamphenicol resistance. The expression of *cas9* was induced by 2.5 mM lactose. After four sequential cultivations in YTF medium supplemented with 4 µg mL^−1^ thiamphenicol plus lactose, the desired knockout event was confirmed by PCR. Anoth﻿er two sequential cultivations in YTF medium without thiamphenicol promoted the loss of the CRISPR plasmid by segregation, followed by plating to obtain single colonies.

### Whole genome sequencing

Whole genome sequencing was carried out by SEQ-IT (Kaiserslautern, Germany) as previously described [35]. The sequence data were analyzed using SeqManPro (DNASTAR, Madison, WI, USA). The contigs were assembled and aligned to the wild-type *C. ljungdahlii* genome (GenBank Accession Number: CP001666.1), the plasmid, and a genomic integration strain created in silico.

### Measurement of cell growth

Samples were drawn with a sterile syringe and cannula through the butyl septum. The optical density was measured at a wavelength of 600 nm (OD_600_) using a BioPhotometer (Eppendorf, Hamburg, Germany). When the OD_600_ value exceeded 0.99, the sample was diluted 1:1 with water for more accurate measurement. A small spatula tip of sodium dithionite was added to the cuvettes as a reducing agent before measuring to decolorize the resazurin in the sample.

### GC–MS analysis

To determine the quantity of alcohols and acids in the cultures, 1 mL of cell suspension was centrifuged (17,000 ×*g*, 2 min, room temperature) and 100 µL of the supernatant was mixed with 900 µL methanol containing 2.2 mM 1,3-propanediol as an internal standard (IS). After centrifugation (17,000 ×*g*, 5 min, room temperature), 750 µL of the supernatant was transferred to glass vials for measurement. GC–MS analysis was carried out by injecting 1 µL with a 1:10 split into a GCMS-QP2010S system (Shimadzu, Kyoto, Japan) fitted with an InertCap FFAP capillary column (0.25 mm × 30 m, 0.25 µm film thickness) from GL Sciences (Eindhoven, Netherlands). The temperature profile started with an initial 3-min hold at 50 °C followed by a gradient of 35 °C min^−1^ to 220 °C with a final hold at 220 °C for 2 min.

### Analysis of CoA-esters

Intracellular metabolites were analyzed as described by Gaida et al. [40] with minor modifications. After resuspension in 500 µL quenching solution, cells were lysed in a bead beater (3 × 30 s) at 4 °C with incubation on ice between pulses. Standard curves were prepared for the different intermediates and octanoyl-CoA served as the internal standard. More details are given in Additional file 1: S1.7 together with the transition list in Additional file 1: Table S6.

### Targeted proteomics

Depending on the growth phase, 15–50 mL of the culture were harvested by centrifugation (4000 ×*g*, 10 min, 4 °C) and the cells were disrupted using a FastPrep 5G Bead Beater (MP Biomedicals Germany, Eschwege, Germany) as described elsewhere [35]. The protein concentration was determined, followed by tryptic digestion and desalting as described elsewhere [40]. Furthermore, a mix containing labeled peptide markers (SpikeTides_L) from JPT Peptide Technologies (Berlin, Germany) was prepared for each peptide to facilitate automatic identification.

For quantitative analysis, calibration curves were prepared from ordered peptides (SpikeTides_TQ, from JPT Peptide Technologies) with a defined amount of 1 nmol. These were dissolved in 100 µL buffer (80% 0.1 M ammonium bicarbonate, 20% acetonitrile), digested with a 1:100 enzyme-to-substrate ratio of trypsin (Promega, Madison, WI, USA) to remove the quantification tag and the standards were purified as previously described [40]. The abundance of each protein of interest was calculated as ng_protein_ µg^−1^_soluble protein_ (in the following: ng µg^−1^).

The samples were separated using an HPLC (Shimadzu Prominence Ultra-Fast Liquid Chromatography) system fitted with a Supelco column (Ascentis express C18) at an oven temperature of 40 °C. Solvent A was 2% acetonitrile and 98% water with 0.1% formic acid, and solvent B was 98% acetonitrile and 2% water with 0.1% formic acid. The column was equilibrated with 5% solvent B and 95% solvent A for 0.1 min before applying a gradient to 40% solvent B in 16.9 min followed by a gradient to 95% solvent B in 0.5 min and a 1.0-min hold. Then a gradient was applied to 5% solvent B in 0.5 min, followed by a 3.0-min hold. A flow rate of 0.4 mL min^−1^ was maintained throughout. The separated peptides were introduced into a 6500 QTRAP MS (AB Sciex, Darmstadt, Germany) by electrospray ionization, and the absolute quantity of the products was determined by multiple reaction monitoring (MRM). More details are provided in Additional file 1: S1.8 together with the transition list in Additional file 1: Table S7.

## Results

### Analysis of *C. kluyveri* fermentation products and intermediates

**Fig. 1:**
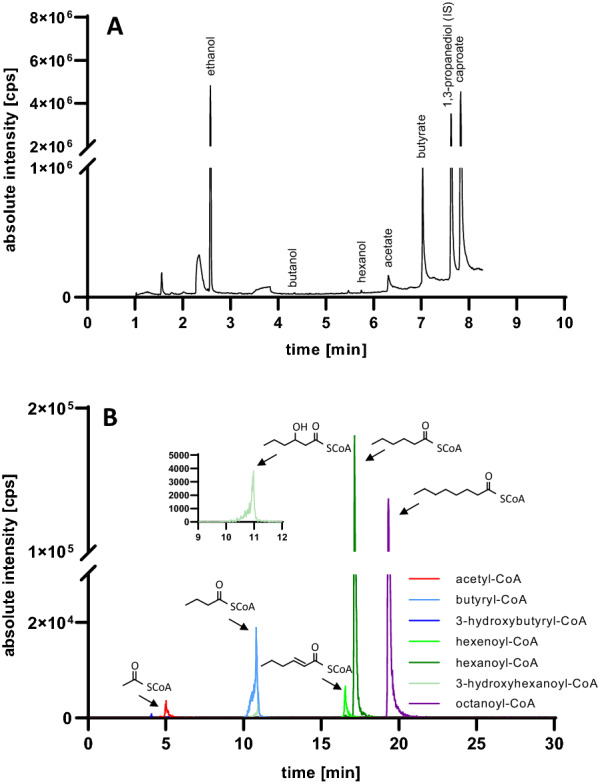
Analysis of fermentation products and CoA-ester intermediates in *C.* *kluyveri*. **A** GC–MS analysis of fermentation broth from a culture of *C.* *kluyveri* revealed the presence of butyrate, caproate, and traces of butanol and hexanol. Ethanol and acetate were derived from the medium and 1,3-propanediol served as the internal standard. **B** A volume of 50 mL of a logarithmically grown *C.* *kluyveri* culture (OD_600nm_ = 0.9) was collected by centrifugation and re-suspended in 500 µL of a quenching solution. The cell debris was removed by centrifugation and sterile filtration. CoA-esters (acetyl-CoA, butyryl-CoA, 3-hydroxybutyryl-CoA, hexenoyl-CoA, hexanoyl-CoA and 3-hydroxylhexanoyl-CoA) were measured by LC–MS/MS with octanoyl-CoA as the internal standard

*Clostridium kluyveri* is a natural producer of caproate (hexanoic acid) and of traces of hexanol from heterotrophic substrates. The biosynthesis of caproate by *C. kluyveri* starts from the central primary metabolite acetyl-CoA. The condensation of two acetyl-CoA molecules yields butyryl-CoA, which is extended by a third acetyl-CoA molecule to form hexanoyl-CoA before conversion to caproate. We evaluated the ability of wild-type *C.* *kluyveri* to form C4 and C6 fermentation products in batch cultivations using serum bottle flasks containing DSM medium 52. We analyzed the fermentation products and intermediates by GC–MS and LC–MS/MS (Fig. 1). *C.* *kluyveri* accumulated 1604 ± 415 mg L^−1^ caproate, 452 ± 161 mg L^−1^ butyrate and 4.6 ± 0.4 mg L^−1^ hexanol in the stationary phase after 11 days of cultivation (n = 2) (Fig. 1A). We also detected ethanol and acetate, which originated from the cultivation medium and were included as carbon and energy sources. In addition, we also analyzed the CoA-esters in *C. kluyveri* cell lysate by LC–MS/MS, revealing concentrations of 29.5 mg L^−1^ for hexanoyl-CoA as the main intermediate, as well as 5.5 mg L^−1^ butyryl-CoA and 2.2 mg L^−1^ acetyl-CoA (Fig. 1B). The accumulation of caproate and hexanoyl-CoA, the CoA-precursor of hexanol, indicated the potential to produce significant amounts of hexanol by expressing a dual-function alcohol dehydrogenase along with the hexanoyl-CoA biosynthesis genes derived from *C.* *kluyveri*.

### Selection of genes for hexanol biosynthesis

Genes potentially involved in the production of caproate were already annotated in the *C.* *kluyveri* genome sequence [41]. The formation of butyryl-CoA is assumed to involve the enzymes acetoacetyl-CoA thiolase (Thl), NAD/NADP-dependent 3-hydroxybutyryl-CoA dehydrogenase (Hbd), 3-hydroxybutyryl-CoA dehydratase (Crt), and the NAD-dependent butyryl-CoA dehydrogenase complex (Bcd/EtfAB). The *C.* *kluyveri* genome encodes at least two versions of these enzymes, and three thiolases. One set (*crt1*, *bcd1*, *etfB1*, *etfA1* and *hbd1*) is located in a single cluster (CKL1072-1078) whereas the other set is dispersed throughout the genome, although *etfB2* and *etfA2* form a tandem pair and the *thlA1*, *thlA2* and *thlA3* genes are also close together. We hypothesized that the large cluster could encode the enzymes required to produce butyryl-CoA and the dispersed set could encode the enzymes required to produce hexanoyl-CoA. The protein sequences of the two sets show only moderate identities of 44% (Crt1 vs Crt2), 55% (Bcd1 vs Bcd2), 44% (EtfB1 vs EtfB2), 47% (EtfA1 vs EtfA2) and 37% (Hbd1 vs Hbd2). The three thiolases are more closely related, with pairwise identities ranging from 79 to 81%. However, the in vivo functions of these enzymes have not yet been investigated experimentally.

The presence of in most cases two homologous genes in the *C.* *kluyveri* genome suggested that each enzyme is responsible for a similar reaction utilizing either a C4 or C6 substrate. We therefore generated a potential hexanol biosynthesis gene cluster containing a gene or gene complex for each of the 10 reactions from acetyl-CoA to hexanol. Given that *C.* *kluyveri* produces large quantities of caproate but only trace amounts of hexanol [24], we omitted the *cat3* gene encoding butyryl-CoA:acetate CoA transferase, which presumably catalyzes the formation of butyrate and caproate, and chose a bifunctional aldehyde-alcohol dehydrogenase from *C.* *acetobutylicum* (AdhE2), which should instead form hexanol, as previously shown in *E. coli* [15].

### Construction of a butanol and hexanol biosynthesis cluster and generation of recombinant C. ljungdahlii

**Fig. 2:**
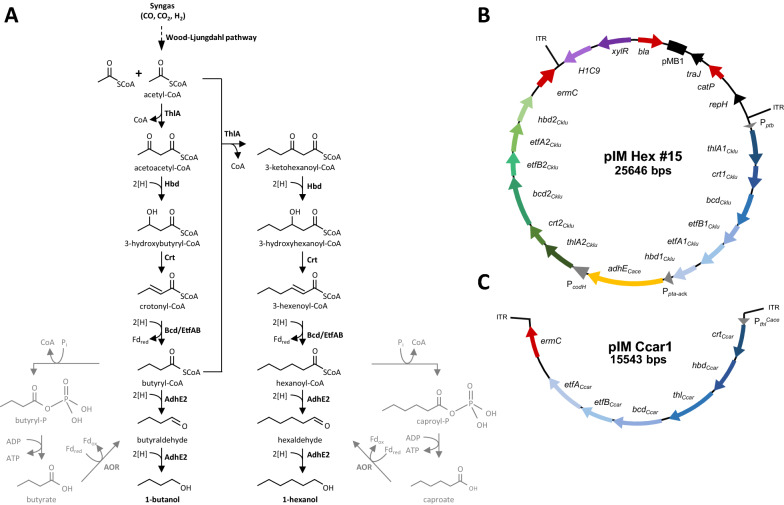
Designed pathway for butanol and hexanol biosynthesis and the corresponding plasmids. **A** Schematic representation of the heterologous butanol and hexanol biosynthesis pathway. ThlA1/2: thiolase A1/2; Hbd1/2: hydroxybutyryl-CoA-dehydrogenase 1/2; Crt1/2: crotonase 1/2; Bcd-EtfA/B complex = Bcd 1/2: butyryl-CoA-dehydrogenase 1/2; EtfA/B 1/2: electron transferring protein A/B 1/2 (all genes from *C.* *kluyveri*); AdhE2: bifunctional aldehyde-alcohol-dehydrogenase from *C.* *acetobutylicum*. Two molecules of acetyl-CoA are condensed by acetyl-CoA acetyltransferase (ThlA) to form acetoacetyl-CoA, which is then reduced to 3-hydroxybutyryl-CoA by 3-hydroxybutyryl-CoA dehydrogenase (Hbd) with NADH as the electron donor. In the next step, 3-hydroxybutyryl-CoA is dehydrated by crotonase (Crt) to form crotonyl-CoA, which is then reduced with NADH and oxidized ferredoxin (Fd) by a Flavin-based electron bifurcating butyryl-CoA dehydrogenase (Bcd) to form butyryl-CoA. From here, butanol can be produced via two reduction steps with NADH as the electron donor, catalyzed by a bifunctional aldehyde dehydrogenase/alcohol dehydrogenase (AdhE2). Alternatively, for the generation of hexanol, a third acetyl group derived from acetyl-CoA is added to the butyryl-CoA to produce 3-ketohexanoyl-CoA. Analogous to the steps in which acetoacetyl-CoA is converted to butyryl-CoA, 3-ketohexanoyl-CoA is again reduced with NADH, dehydrated, and reduced again by a bifurcating butyryl-CoA dehydrogenase to form hexanoyl-CoA. A bifunctional aldehyde dehydrogenase/alcohol dehydrogenase then catalyzes the final reduction steps to form hexanol, with NADH as the electron donor. Butyrate and caproate are potential side products and can be derived from butyryl-CoA and hexanoyl-CoA, respectively, with butyryl phosphate and caproyl phosphate as the corresponding intermediates. After reduction of the acids butyrate and caproate to the respective aldehydes via the native AOR (aldehyde:ferredoxin oxidoreductase) the molecules can be further converted to the alcohols butanol and hexanol. These alternative pathways allow the conservation of energy in form of ATP production via substrate level phosphorylation and are shown in grey (adapted from [26]) **B** Schematic representation of pIM Hex#15. The integration cassette consisting of a butanol-hexanol biosynthesis cluster and the adjacent *ermC* sequence is flanked by the mycomar sites (ITR–inverted terminal repeats) which allow integration catalyzed by the xylose-inducible Himar1 transposase. **C** Schematic representation of the Ccar1 cassette with the butanol/ hexanol biosynthesis cluster of *C.* *carboxidivorans* (pIM Ccar1)

The *C. kluyveri* genes proposed to be necessary for butyryl-CoA and hexanoyl-CoA production and the bifunctional aldehyde-alcohol dehydrogenase from *C.* *acetobutylicum* were combined in vector pIM Hex#15 (Fig. 2). The genes were grouped into two operons: one containing *thlA1, crt1, bcd1, etfB1, etfA1* and *hbd1* under the control of the *C.* *acetobutylicum* phosphate butyryl transferase (*ptb*) promoter, and the other containing *thlA2, crt2, bcd2, etfB2, etfA2* and *hbd2* under the control of the *C.* *ljungdahlii* CO-dehydrogenase (*codH*) promoter. Furthermore, the *adhE2* gene was placed under the control of *C. ljungdahlii* phosphotransacetylase/acetate kinase (*pta-ack*) promoter (see Table S2, Fig. S1 and Fig. S2 in Additional file 1: S1.3).

**Fig. 3:**
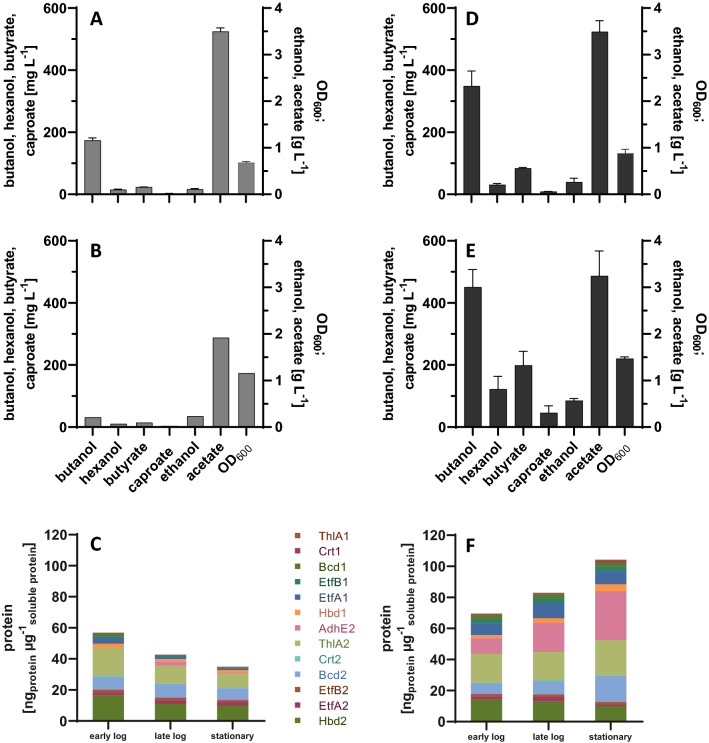
Comparison of plasmid-carrying and genomic integration strains. Maximum product concentrations and optical densities achieved by *C.* *lju* pIM Hex#15 (**A**, **B**) and *C.* *lju* Hex#15^gInt^ (**D**, **E**) in serum bottle cultivations (**A**, **D**) and 2-L fermentations (**B**, **E**) with ATCC 1754 medium continuously supplied with 20% CO_2_ and 80% H_2_ as the carbon and energy sources. Data are means ± SD (n = 3 biological replicates, data in B: n = 1). Enzyme concentrations as determined by LC–MS/MS from *C.* *lju* pIM Hex#15 (C) and *C.* *lju* Hex#15^gInt^ (F) in the early logarithmic (OD_600_ ~ 0.3–0.5), late logarithmic (OD_600_ ~ 0.5–1.0) and stationary phases (OD_600_ > 1.0) in a 2-L fermentation

The pIM Hex#15 vector was introduced into *C. ljungdahlii* by conjugation to yield strain *C.* *lju* pIM Hex#15. Single colonies were selected and analyzed for their ability to produce butanol and hexanol. Titers of 1075 ± 324 mg L^−1^ butanol and 133 ± 18 mg L^−1^ hexanol (n = 3) were obtained after 120 h when grown in YTF medium using fructose as carbon source. In order to investigate product formation on different gaseous substrates in minimal medium, the heterologous strain was cultivated on CO-containing gas (33% CO, 33% CO_2_, 33% H_2_) and on CO_2_ as carbon source (20% CO_2_, 80% H_2_). Butanol and hexanol were successfully produced with both gas compositions, with significantly higher production titers on 20% CO_2_ and 80% H_2_ (see Fig. S3 in Additional file 1: S2.1). Based on these preliminary results, we focused on the utilization of carbon dioxide in further experiments. Starting with serum bottle cultivation supplied with 20% CO_2_ and 80% H_2_, *C.* *lju* pIM Hex#15 produced 174 ± 8 mg L^−1^ butanol and 15 ± 2 mg L^−1^ hexanol after 220 h, reaching an OD_600_ of 0.68 ± 0.03 (Fig. 3A). When analyzing protein samples from strain *C.* *lju* pIM Hex#15 all enzymes encoded on the plasmid were detected by targeted proteomics, confirming the expression of all genes in the heterologous biosynthesis clusters (data not shown).

Next, we integrated the functional gene cluster into the *C. ljungdahlii* genome in order to avoid problems caused by plasmid loss, allowing fermentation without continuous selection for antibiotic resistance. To facilitate integration, the pIM vector is equipped with a xylose-inducible transposase system [35]. We therefore supplemented the culture of *C. lju* Hex#15 with 2% (w/v) d-xylose in order to induce the Himar1 transposase. This is thought to promote integration at random sites containing the dinucleotide TA [42]. Genomic integration of the 17.9-kb cluster resulted in strain *C.* *lju* Hex#15^gInt^ and the site of integration was identified by inverse PCR. Closer analysis of one positive clone revealed an integration event at position 364,132 of the *C.* *ljungdahlii* genome (CP001666.1) between the genes CLJU_c03490 and CLJU_c03500, both encoding hypothetical conserved proteins.

The integration site was verified by PCR over the junction sites between the inserted cluster and the genomic flanking sequences (as shown in Fig S4 in Additional file 1: S2.2). The position and integrity of the cluster was confirmed by whole genome sequencing. Repetitive inoculation of the isolated strain in YTF medium led to the loss of the pIM Hex#15 plasmid, which was confirmed by (1) the absence of plasmid DNA in the whole genome sequencing data and (2) the response of the strain to antibiotic selection. Growth in the presence of clarithromycin alone confirmed the genomic integration of the *ermC* gene, and the absence of growth in the presence of thiamphenicol indicated the loss of the *catP* gene located on the plasmid backbone.

### Characterization of strain *C. lju* Hex#15^gInt^

The heterologous production of butanol and hexanol was compared between the genomic integration strain *C.* *lju* Hex#15^gInt^ (carrying a single integrated copy of the gene cluster) and the plasmid bearing strain *C.* *lju* pIM Hex#15 (supposed to carry multiple copies of the pIM Hex#15 plasmid) in serum bottle cultures and fermentations with a continuous gas supply. *C.* *lju* Hex#15^gInt^ produced twice as much butanol and hexanol as the plasmid-carrying strain in serum bottles after 143 h: 349 ± 48 mg L^−1^ vs 174 ± 8 mg L^−1^ butanol and 31 ± 4 mg L^−1^ vs 15 ± 2 mg L^−1^ hexanol (Fig. 3A, D). *C.* *lju* Hex#15^gInt^ also produced larger quantities of other fermentation products such as butyrate (84 ± 3 mg L^−1^ vs 23 ± 1 mg L^−1^), caproate (9 ± 0 mg L^−1^ vs 3 ± 0 mg L^−1^) and produced also more biomass (OD_600_ at 0.88 ± 0.09 vs 0.68 ± 0.03).

The difference in performance between the plasmid-carrying and genomic integration strains became even more evident in 2-L fermentations, where controlled cultivation conditions can be maintained (Fig. 3B, E). In contrast to the serum bottle cultures, the pH was maintained at 6.0 during fermentation and CO_2_ and H_2_ were supplied continuously. The plasmid-carrying strain *C.* *lju* pIM Hex#15 produced 31 mg L^−1^ butanol, 10 mg L^−1^ hexanol, 14 mg L^−1^ butyrate, and 3 mg L^−1^ caproate and reached an OD_600_ of 1.16 in minimal medium supplemented with 4 mg L^−1^ clarithromycin to maintain the plasmid. In contrast, *C.* *lju* Hex#15^gInt^ produced 451 ± 56 mg L^−1^ butanol, 122 ± 41 mg L^−1^ hexanol, 199 ± 45 mg L^−1^ butyrate, 46 ± 23 mg L^−1^ caproate and reached an OD_600_ of 1.47 ± 0.04 in medium without antibiotics (Fig. 3B, E). The genomic integration strain *C.* *lju* Hex#15^gInt^ therefore accumulated 15-fold more butanol and 12-fold more hexanol than the *C. lju* pIM Hex#15 strain, containing the gene cluster still on the used plasmid. Furthermore, the fermentation of *C.* *lju* Hex#15^gInt^ at 2-L scale produced 1.3-fold more butanol, 3.9-fold more hexanol, 2.4-fold more butyrate, 5.6-fold more caproate and 2.2-fold more ethanol than the same strain in serum bottles, and the OD_600_ was 67% higher, but the acetate concentration was similar for both systems.

In order to exclude an effect of the used antibiotic clarithromycin we compared the gas fermentation of the strains *C.* *lju* pIM Hex#15 and *C.* *lju* Hex#15^gInt^ in medium supplemented with clarithromycin. *C.* *lju* Hex#15^gInt^ produced less butanol and hexanol than in the fermentations without clarithromycin, but still fivefold higher titers of butanol and eightfold higher titers of hexanol than *C.* *lju* pIM Hex#15 even though the genomic integration strain carried only a single copy of the gene cluster. We therefore examined the effect of gene dosage on enzyme expression levels by targeted proteomics.

We detected all 13 proteins encoded by the gene cluster in both strains (Fig. 3C, F). Detailed results of the targeted proteomics analysis are listed in Table S10 in Additional file 1: S2.5. The levels of AdhE2, ThlA2, Bcd2 and Hbd2 were comparable to those of native Wood-Ljungdahl pathway enzymes such as AcsB when normalized to the total soluble protein (all > 10 ng µg^−1^), whereas all other heterologous enzymes were less abundant (< 5 ng µg^−1^). Comparing the two strains during the stationary phase, the enzymes ThlA1, Crt1, EtfA1, ThlA2, Bcd2 and AdhE2 were more abundant in strain *C.* *lju* Hex#15^gInt^ with a single integrated copy of the gene cluster. However, we observed a clear diverging trend in protein abundance over time in the two strains (Fig. 3C, F). Starting from a similar total quantity of all 13 enzymes (normalized to total soluble protein) in both strains during the early log phase, the enzyme abundance dropped by 39% in the plasmid-carrying strain but increased by 50% in the genomic integration strain as fermentation progressed. Coupling these findings to the product concentrations indicated that the higher rate of product formation in strain *C.* *lju* Hex#15^gInt^ may correlate with enzyme abundance. This suggested that the limited availability of one or more enzymes could result in a bottle-neck affecting product yields.

We found that heterologous enzymes under the control of the *C.* *acetobutylicum ptb* promoter were 2.5-fold less abundant than those controlled by the *C. ljungdahlii codH* promoter in the genomic integration strain, representing a potential bottle-neck for butanol and also hexanol biosynthesis. We hypothesized that increasing the expression of enzymes responsible for butyryl-CoA production could enhance the formation of butanol and hexanol. This could either be achieved by exchanging the promoter of the lower expressed operon against a stronger promoter or by introducing an additional gene cluster. We decided to introduce additional enzymes from *C.* *carboxidivorans* that catalyze the formation of butyryl-CoA, but this required the removal of the selectable marker *ermC* from the *C.* *lju* Hex#15^gInt^ genome due to the limited availability of selection markers for *C.* *ljungdahlii*.

### Excision of ermC selection marker using CRISPR/Cas9

The 1236-bp *ermC* gene cassette coding for the clarithromycin resistance marker was removed from strain *C.* *lju* Hex#15^gInt^ using the CRISPR/Cas9 system so that the same marker could be used in the next round of transformation. We therefore designed a plasmid carrying sgRNA targeting the *ermC* gene, a lactose-inducible *cas9* gene, homology arms to promote homology-dependent repair of the *ermC* locus, and *catP* as the selectable marker. Following the transformation of *C.* *lju* Hex#15^gInt^ with pCJRK Δ*ermC*, *cas9* expression was induced by four consecutive inoculation steps with 2.5 mM lactose. The excision of the *ermC* gene was verified by the amplification of genomic DNA from single colonies (as shown in Figure S5 in Additional file 1: S2.3) resulting in the isolation of strain *C.* *lju* Hex#15^gInt^ Δ*ermC*, which was analyzed further by whole genome sequencing and does not contain any selection markers.

### Introduction of additional pathway genes

We addressed the low abundance of butanol/hexanol pathway enzymes like ThlA1 in strain *C.* *lju* Hex#15^gInt^ Δ*ermC* by introducing the *C.* *carboxidivorans* genes *crt*, *hbd*, *thl*, *bcd*, *etfB* and *etfA* for butyryl-CoA/hexanoyl-CoA biosynthesis. Two homologous sets of these genes are present in the *C.* *carboxidivorans* genome: Ccar_RS22775 – Ccar_RS22800 (Ccar1) and Ccar_RS01400 – Ccar_RS01430 (Ccar2). We therefore amplified them from genomic DNA and placed them under the control of the *C.* *acetobutylicum* constitutive *thlA* promoter or the *C.* *ljungdahlii* constitutive *codH* promoter in an *E.* *coli*–*C.* *ljungdahlii* shuttle vector. The resulting plasmids (pIM Ccar1 and pIM Ccar2) were introduced into wild-type *C.* *ljungdahlii*. Butanol and hexanol formation was only detectable in the strain carrying pIM Ccar1 (data shown in Figure S6 in Additional file 1: S2.4). We therefore introduced pIM Ccar1 into *C.* *lju* Hex#15^gInt^ Δ*ermC* by conjugation, resulting in strain *C.* *lju* Hex#15^gInt^ pIM Ccar1, which produced 150 mg L^−1^ butanol and 450 mg L^−1^ hexanol on YTF medium. The Ccar1 cluster was then integrated into the genome as described earlier for the pIM Hex#15 cluster to ensure stable expression in the absence of antibiotics. The resulting double genomic integration strain was named *C.* *lju* Hex#15^gInt^ Ccar1^gInt^. The cluster was integrated at position 4,355,156 between the genes CLJU_c40040 and CLJU_c40050, encoding two hypothetical proteins. The double genomic integration strain was then compared to the single genomic integration strain for growth and product formation on 20% CO_2_ and 80% H_2_.

**Fig. 4:**
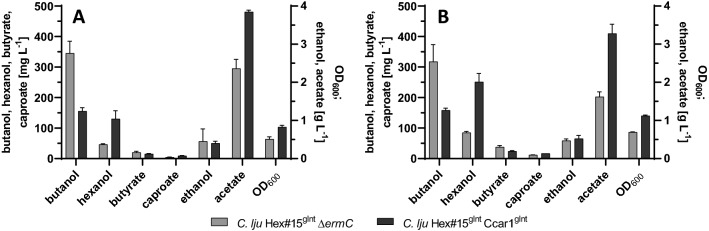
Maximum product concentrations and optical densities achieved during the stationary phase with strains *C.* *lju* Hex#15^gInt^ Δ*ermC* and *C.* *lju* Hex#15^gInt^ Ccar1^gInt^ at 37 °C (**A**) and 30 °C (**B**) in 125-mL serum bottles containing 25 mL ATCC 1754 medium supplied with 20% CO_2_ and 80% H_2_ as the carbon and energy sources. Data are means ± SD (n = 3 biological replicates)

The introduction of the additional *C.* *carboxidivorans* genes improved the yields of hexanol (2.3-fold increase) and caproate (1.8-fold increase) at the expense of butanol (54% decrease) and butyrate (25% decrease) at 37 °C (Fig. 4A). Given that temperatures below 37 °C improve alcohol production by *C.* *carboxidivorans* [25, 26], we also cultivated the single and double genomic integration strains at 30 °C (Fig. 4B). The yield of butanol did not change significantly at the lower temperature in strain *C.* *lju* Hex#15^gInt^ Δ*ermC* (345 ± 40 mg L^−1^ at 37 °C vs 318 ± 56 mg L^−1^ at 30 °C) or strain *C.* *lju* Hex#15^gInt^ Ccar1^gInt^ (155 ± 12 mg L^−1^ at 37 °C vs 158 ± 7 mg L^−1^ at 30 °C). However, hexanol formation at 30 °C increased 1.8-fold in the single genomic integration strain and 2.0-fold in the double genomic integration strain. Accordingly, the highest titer of hexanol we achieved was 251 ± 28 mg L^−1^ by cultivating the double genomic integration strain *C.* *lju* Hex#15^gInt^ Ccar1^gInt^ at 30 °C in serum bottles. Compared to the original plasmid-carrying strain *C.* *lju* pIM Hex#15, the double genomic integration strain produced 15 times as much hexanol in serum bottles fed with 20% CO_2_ and 80% H_2_, but there was no significant change in the yield of butanol.

**Fig. 5:**
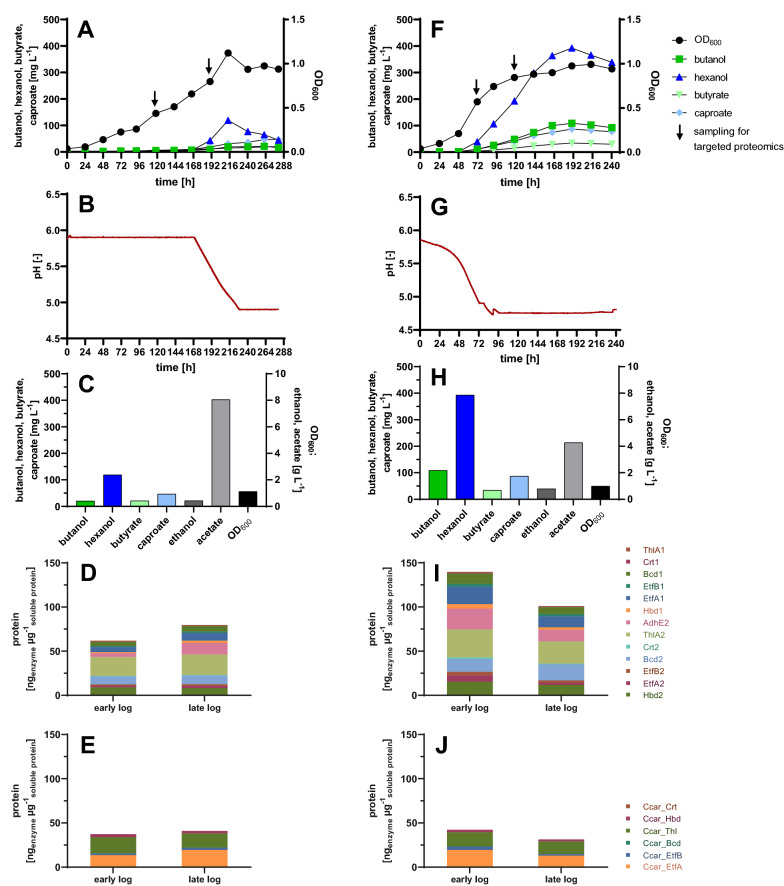
Fermentation of *C. lju* Hex#15^gInt^ Ccar1^gInt^ at 30 °C in ATCC 1754 medium at 2-L scale with a continuous supply of 20% CO_2_ and 80% H_2_ as the carbon and energy sources. **A**–**E** Fermentation with pH control (pH 6.0). **F**–**J** Fermentation without initial pH control. **A**, **F** Fermentation course showing the formation of biomass and heterologous products over time. **B**, **G** The pH of the fermentation broth plotted over time. **C**, **H** Maximum product concentrations and optical densities. **D**, **I** Abundance of heterologous enzymes encoded by the Hex#15 construct. **E**, **J** Abundance of heterologous enzymes encoded by the Ccar1 construct

In the fermentation vessels with a continuous gas supply and pH control at 30 °C, the double genomic integration strain did not produce any butanol or hexanol until 168 h of process time had elapsed, although growth and acetate formation were detected (Fig. 5A). At that point, pH regulation was stopped to allow natural acidification (Fig. 5B). Butanol and hexanol biosynthesis started immediately afterwards and reached maximum concentrations of 20 mg L^−1^ and 119 mg L^−1^, respectively (Fig. 5C). The maximum hexanol concentration was below that achieved in serum bottles (251 mg L^−1^) probably due to the late onset of production. We therefore initiated another round of fermentation, this time without initial pH regulation to mimic the conditions in serum bottles, although we ensured that the pH did not drop below pH 4.75 (Fig. 5G). As anticipated, butanol and hexanol formation began earlier under these conditions, between 48 and 72 h (Fig. 5F). Accordingly, the double genomic integration strain produced 109 mg L^−1^ butanol and 393 mg L^−1^ hexanol. Bacterial growth was linear with a constant pH 6.0 (Fig. 5A), whereas faster, logarithmic growth was observed in the absence of pH regulation (Fig. 5F), although in both cases the final OD_600_ was ~ 1.0.

The abundance of the heterologous enzymes in the double genomic integration strain was assessed again by targeted proteomics during fermentations with pH control and without (Fig. 5D, E, I, J). The 13 enzymes encoded by the Hex#15 construct were more abundant in the fermentation without pH regulation (14.0% and 10.1% of the total soluble protein during the early and late logarithmic growth phase, respectively) than in the pH-regulated fermentation run (6.2% and 8.0% of the total soluble protein) (Fig. 5D, I). In contrast, the six enzymes encoded by the Ccar1 construct were rather equally abundant in the early logarithmic growth phase, representing 3.7% of the total soluble protein in presence of pH control and 4.2% of the total soluble protein without pH control (Fig. 5E, J). The most abundant heterologous enzyme encoded by the Ccar1 cassette in strain *C.* *lju* Hex#15^gInt^ Ccar1^gInt^ was Ccar_Thl (14.24–18.23 ng µg^−1^), comparable to the native Wood-Ljungdahl pathway enzyme Fhs (Formate-tetrahydrofolate ligase) (11.44–21.57 ng µg^−1^). Detailed results of the targeted proteomics analysis are listed in Table S11 in Additional file 1: S2.6.

## Discussion

The sustainable production of hexanol and butanol offers a promising alternative to fossil fuels. Several *Clostridium* species can synthesize hexanol and butanol, in some cases even from gaseous substrates and metabolic engineering of genetically amenable strains can be used to increase productivity. We therefore introduced 13 genes for butanol and hexanol biosynthesis from related *Clostridium* species into *C.* *ljungdahlii* as a 17.9-kb gene cluster, facilitating the production of 174 ± 8 mg L^−1^ butanol and 15 ± 2 mg L^−1^ hexanol in serum bottles with 20% CO_2_ and 80% H_2_ as the substrate (Fig. 3A). The integration of this cluster into the *C.* *ljungdahlii* genome increased the butanol yield to 349 ± 48 mg L^−1^ and the hexanol yield to 31 ± 4 mg L^−1^ (Fig. 3D). When scaled up to a 2-L fermentation with continuous gas feeding, the difference between the plasmid-carrying strain *C.* *lju* pIM Hex#15 and the genomic integration strain *C.* *lju* Hex#15^gInt^ was even more pronounced (Fig. 3B, E). To the best of our knowledge, this is the first report describing the heterologous production of hexanol from a gaseous substrate in a *Clostridium* species.

The genomic integration strain *C.* *lju* Hex#15^gInt^ carried a single copy of the butanol/hexanol gene cluster whereas the plasmid-carrying strain *C.* *lju* pIM Hex#15 contained presumably multiple copies of the plasmid. The superior performance of strain *C. lju* Hex#15^glnt^ may reflect the metabolic burden caused by the excessive transcription of plasmid-borne gene copies, as previously reported [43]. We therefore used targeted proteomics to quantify the corresponding enzymes, revealing that all 13 were expressed and accounted for 57–70 ng per µg total soluble protein in the early growth phase, increasing to 100 ng µg^−1^ (10% of the total soluble protein) in strain *C.* *lju* Hex#15^gInt^ during the stationary phase. The abundance of the heterologous enzymes during the fermentation declined by 39% in *C.* *lju* pIM Hex#15 but increased by 50% in *C.* *lju* Hex#15^gInt^ (Fig. 3C, F), confirming that a single integrated copy outperforms multiple episomal copies of the same genes. A study performed in *E. coli* showed that the expression of the fluorescent protein mCherry under the control of an inducible promoter is depending on the location in the genome [44]. After integration of the respective gene at four different genome positions, the measured fluorescence level varied from 25 to 500% compared to cultures with the gene expressed on a high-copy plasmid. Hence, not only the copy number, but also the location of the integration most likely influences the expression of the heterologous pathway in *C. ljungdahlii.* In our study, both genomic integration events resulted in an insertion between two hypothetical proteins leaving the coding sequences intact. Analysis of the expression and product levels of strains with different and multiple sites of integrations would be interesting and should offer improvements in product titers.

The native enzyme AcsB (acetyl-CoA synthase of the CO dehydrogenase/acetyl CoA synthase complex beta subunit, CLJU_c37550) is part of the Wood-Ljungdahl pathway and was used as an internal control, and it represented between 3.3% and 6.8% of total soluble protein in the two producer strains under autotrophic growth conditions. This is in a similar range as reported by Richter et al*.* [45], who measured the abundance of AcsB in *C.* *ljungdahlii* on CO-rich substrate to be 1.94% of the total protein. Heterologous enzymes for the production of butanol in *C.* *cellulolyticum* each covered 0.15–2% of cytosolic protein [40], being in line with the here reported heterologous enzyme abundance.

We hypothesized that the lower product concentrations achieved by the plasmid strain *C.* *lju* pIM Hex#15 reflected the limited abundance of the corresponding enzymes during later fermentation stages and developed a strategy to increase enzyme abundance in strain *C.* *lju* Hex#15^gInt^ to improve its performance even further. Therefore, we removed the 1236-bp *ermC* selection marker using the CRISPR/Cas9 system, which has already been used in *C.* *ljungdahlii* as a rapid and efficient strategy to remove genes as large as 2.6 kb [37]. For further strain development, CRISPR/Cas9 could also be used to remove genes promoting the formation of side products and/or to insert or exchange regulatory sequences such as promoters [37]. Promoter engineering in *C.* *acetobutylicum* mostly resulted in weaker or only slightly improved promoters, whereas optimization of ribosome binding site (RBS) and start codon was more efficient, leading to more than tenfold increased expression [46]. As we wanted to increase the expression of an operon encoding for six enzymes, the straight forward strategy to achieve higher expression of all genes/enzymes was to introduce an additional construct instead of engineering the RBS of each gene.

We subsequently added the butanol (butyryl-CoA) gene cluster from *C.* *carboxidivorans* [27] under the control of the constitutive *thlA* promoter from *C.* *acetobutylicum* [37]. The resulting double genomic integration strain *C.* *lju* Hex#15^gInt^ Ccar1^gInt^ achieved a significant increase in hexanol formation, reaching titers of 251 ± 28 mg L^−1^ when cultivated with 20% CO_2_ and 80% H_2_ as a substrate (Fig. 4B). The assigned function of the *C.* *carboxidivorans* operon Ccar_RS22775 – Ccar_RS22800 (Ccar1) is butanol production [27] so we assumed that the closely related *C. carboxidivorans* operon Ccar_RS01400 – Ccar_RS01430 (Ccar2) would be responsible for native hexanol production. However, when we tested each operon by transferring them into wild-type *C. ljungdahlii*, we found that the Ccar1 operon controlled by the *C. acetobutylicum thlA* promoter led to the production of both butanol and hexanol, whereas the Ccar2 operon controlled by the *C.* *ljungdahlii codH* promoter led to the production of neither. During the course of our work, Wirth and Dürre [16] published a more closer examination of genes involved in the biosynthesis of hexanol also including the genes from *C.* *carboxidivorans*. According to their work, only Ccar1 is transcribed during the heterotrophic growth of *C.* *carboxidivorans - *no Ccar2 transcripts were detected. The C6 molecule caproate could be detected in autotrophically grown *A.* *woodii* cultures harboring either the Ccar1 or Ccar2 cluster on a plasmid [16]. It therefore seems unlikely that the two *C.* *carboxidivorans* operons are strictly associated with separate products. Instead, Ccar1 appears to be the only active cluster under the heterotrophic cultivation conditions tested thus far. Expression of the Ccar2 cluster could be triggered under different metabolic conditions (carbon and nutrient supply) and/or different from Ccar1, cluster Ccar2 could use specifically C4 substrates. The genomic integration of the Ccar1 cluster into the heterologous producer strain *C.* *lju* Hex#15^gInt^ Δ*ermC* increased hexanol formation at the expense of butanol. This might be the result of the high abundance of the enzyme Ccar_Thl (accounting for 1.4–1.8% of the total soluble protein in *C.* *lju* Hex#15^gInt^ Ccar1^gInt^), which is assumed to be responsible for chain elongation by acetylating two C2 molecules (acetyl-CoA) to a C4 molecule (acetoacetyl-CoA), or one C4 (butyryl-CoA) with one C2 (acetyl-CoA) molecule to a C6 molecule (3-ketohexanoyl-CoA). Alternatively, butyryl-CoA and hexanoyl-CoA can be reduced to butanol and hexanol, respectively by the similarly abundant heterologously expressed *C. acetobutylicum* alcohol dehydrogenase [15] or via the native *C.* *ljungdahlii* AdhE or AOR (aldehyde:ferredoxin oxidoreductase) [47]. Albeit only native AOR and not AdhE (native) was found to be present in a significant amount under autotrophic conditions in *C. ljungdahlii* [45]. The product ratio of butanol to hexanol was shifted from ~ 5:1 in *C.* *lju* Hex#15^gInt^ to ~ 1:2 in *C.* *lju* Hex#15^gInt^ Ccar1^gInt^, favoring the accumulation of the longer-chain alcohol. However, the first fermentation run with *C.* *lju* Hex#15^gInt^ Ccar1^gInt^ produced lower butanol and hexanol titers than serum bottle cultivations with the same strain (Fig. 5C and Fig. 4B). One potential explanation is the different pH profile in the two systems. In serum bottles, the pH declines as acetate is produced and drops to pH ~ 4, which results in growth arrest. In the fermentation, we therefore maintained a constant pH of 6.0. However, no alcohol formation was detected until 168 h, so the titration with NaOH was stopped and natural acidification was allowed to take place, similar to the serum bottle culture (Fig. 5B). The formation of butanol and hexanol started as soon as we stopped adding NaOH, although titration was restarted when the pH fell to 4.85 to avoid an acid crash [48, 49]. Our results support the important impact of the AOR for alcohol formation in *C. ljungdahlii*. Previous studies hypothesized, that due to the lower pH a higher intracellular concentration of undissociated acids is available as substrate for the AOR [45] and AOR can accept a variety of carboxylic acids (acetate, butyrate, caproate) and convert these to their corresponding aldehydes [50].

The late formation of butanol and hexanol limited the maximum concentration, but this was addressed by initiating fermentation with the double genomic integration strain without initial pH regulation (Fig. 5G). The maximum hexanol concentration we achieved under these conditions was 393 mg L^−1^ (Fig. 5H). This result confirms that the product spectrum of *C.* *ljungdahlii* growing on syngas can be extended to include longer-chain alcohols by introducing new biosynthesis pathways. The heterologous production of hexanol has already been reported in *E. coli* with glucose as the carbon source, reaching titers of 47 mg L^−1^ [15] and 469 mg L^−1^ [51] and in *C.* *saccharoperbutylacetonicum* with glucose as the carbon source, reaching a titer of 531 mg L^−1^ [16]. An alternative approach is the co-cultivation of an acetogenic (syngas-consuming) strain and a solventogenic (solvent-producing) strain, the former to metabolize the gaseous substrate and produce mainly ethanol and acetate, and the latter to use these as substrates to produce alcohols such as butanol and hexanol, or their precursors [31]. For example, co-cultures of *C.* *kluyveri* and *C.* *ljungdahlii* produced 726 mg L^−1^ d^−1^ butanol and 540 mg L^−1^ d^−1^ hexanol in continuous syngas fermentation [31]. *C.* *ljungdahlii* was able to achieve the heterologous production of ~ 150 mg L^−1^ butanol, but it was largely converted to butyrate later during cultivation [29], possibly reflecting the activity of native butanol dehydrogenase [52] or reverse reaction of the native AOR enzyme [50]. Our producer strains also formed small amounts of butyrate, but the butanol concentration remained stable at the end of cultivation. This indicates that the activity of dehydrogenases or reductases remains low under our cultivation conditions without pH regulation and in the presence of 20% CO_2_ and 80% H_2_.

Thus far, *C. ljungdahlii* has been shown to accumulate only low levels of heterologous products when grown on syngas or CO_2_ and H_2_, including acetone at 35 mg L^−1^ [35] and 810 mg L^−1^ [38], isopropanol at 84 mg L^−1^ [53] and 140 mg L^−1^ [35], mevalonate at 68 mg L^−1^ and isoprene at ~ 2 µg L^−1^ [54]. Weitz et al*.* [55] were able to increase the isobutanol yield from 30 mg L^−1^ to 74 mg L^−1^ by feeding with the precursor isovalerate. Woolston et al*.* [56] reported the autotropic production of 3-hydroxybutyrate with a yield of 47 mg L^−1^. The heterologous product with the highest titers reported so far is butyrate, with a yield of ~ 1500 mg L^−1^ [57] and 1004 mg L^−1^ [58]. In the latter study, the simultaneous formation of ~ 200 mg L^−1^ butanol was also reported, suggesting that *C.* *ljungdahlii* can convert butyryl-CoA and/or butyrate to butanol using native enzymes that catalyze the reduction of acetyl-CoA to ethanol [45, 47, 50, 58]. The high yields of butyrate may reflect the involvement of a butyrate kinase, which conserves ATP and increases the yield to > 1 g L^−1^. The other heterologous pathways are not directly involved in energy conservation, explaining the lower yields [59]. Katsyv and Müller [60] characterized the energetics of *C.* *autoethanogenum* growing on CO_2_ and H_2_ or CO and concluded that butanol formation on CO_2_ and H_2_ would only be energetically possible if bifurcating enzymes are involved to couple endergonic and exergonic reactions. A prominent example is the *C. kluyveri* Bcd/EtfAB complex, which is included on vector pIM Hex#15. The endergonic reduction of ferredoxin with NADH is coupled to the exergonic reduction of crotonyl-CoA to butyryl-CoA [61, 62]. These reactions yield NAD^+^ and reduced ferredoxin, which are used to pump protons over the cell membrane with the Rnf complex, driving ATP conservation via a proton gradient [63]. The *C.* *ljungdahlii* Rnf complex couples the electron transfer from reduced ferredoxin to NAD^+^ with the translocation of two protons over the membrane. Four protons are then used to conserve energy as ATP via an ATPase [64]. Using this mechanism, 0.5 ATP/butyryl-CoA units can be conserved for every molecule of acetyl-CoA. In our *C.* *ljungdahlii* producer strain, this means that an additional 0.5 ATP/butanol and 0.75 ATP/hexanol units can be conserved by using the bifurcating Bcd/EtfAB enzyme complex to release one reduced ferredoxin together with two molecules of NAD^+^ [61]. Furthermore, the formation of butanol and hexanol requires four and six NADH molecules, respectively, and can therefore serve as a sink for reducing equivalents.

The product concentrations we achieved are not yet industrially relevant. However, the introduction of two non-optimized “stitched together” biosynthetic gene clusters in *C. ljungdahlii* has already achieved hexanol yields that, when normalized to biomass, are comparable to natural hexanol producers such as *C. carboxidivorans*. The maximum hexanol concentration reported for *C.* *carboxidivorans* on CO-rich syngas was 2442 mg L^−1^ at an OD_600_ of 6.2 (394 mg L^−1^ OD_600_^−1^). The maximum hexanol concentration we achieved by growing the engineered *C. ljungdahlii* strain on CO_2_ and H_2_ was 393 mg L^−1^ at an OD_600_ of 0.99 (397 mg L^−1^ OD_600_^−1^). An OD_600_ of ~ 1 was also reported for wild-type *C.* *ljungdahlii* growing on CO_2_ and H_2_ [57, 65, 66]. Increasing biomass formation, for example by optimizing the medium composition, pH and fermentation setup, could therefore increase butanol and hexanol concentration even further. Li et al*.* [67] reported a 7.6-fold increase in butanol and 44-fold increase in hexanol concentrations (~ 0.1 g L^−1^ hexanol) and doubled the OD_600_ of *C.* *carboxidivorans* by increasing the amount of Zn^2+^ in the medium from 7 to 280 µM, probably caused by an increased gene expression of carbon fixation and alcohol dehydrogenase. In situ product removal to circumvent product toxicity, *e.g.* by gas-stripping [31] or the addition of oleyl alcohol for liquid–liquid extraction [26], was shown to improve hexanol production. Higher fermentation pressure can also increase product yields because the solubility of the energy source H_2_ is low (1.5 mg L^−1^ at 30 °C) compared to the carbon source CO_2_ (1320 mg L^−1^ at 30 °C) and the higher pressure improves H_2_ solubility. However, increasing the pressure of the substrate gas (CO_2_ and H_2_) to 4 and 7 bar inhibited the growth of *C.* *ljungdahlii*, accompanied by a decrease in acetate levels and the accumulation of formate [68, 69]. The same effect was also observed in *A. woodii* with elevated H_2_ partial pressures [70]. Further pathway engineering and classical strain improvement approaches should also make further gains feasible. Genes in the integrated clusters can be replaced with those encoding more active enzymes. For example, Machado et al*.* [51] achieved a 10-fold increase in hexanol production by replacing the *C.* *acetobutylicum* enzyme Hbd with *Ralstonia eutropha* PaaH1 in their cluster for hexanol production in *E. coli*. The exchange or modification of promoters or ribosome binding sites can also increase product formation by modulating transcription and protein synthesis [46, 57]. In a very recent study by Liew et al*.* variation of the promoter alone led to an 11-fold improvement in production when optimizing the acetone biosynthesis pathway for expression in *C. autoethanogenum* [71]*.*

Alternatively, CRISPR/Cas9 can be used to knock out competing pathways leading to byproducts such as acetate or ethanol. However, when deleting the *pta* gene in wild-type *C.* *ljungdahlii*, Huang et al*.* [37] observed significantly impaired growth accompanied by reduced acetate and ethanol formation. The deletion of the *C.* *ljungdahlii* *adhE1* gene significantly reduced ethanol production whereas growth and acetate formation under autotrophic conditions were comparable to wild-type strain [37]. In contrast, Lo et al*.* [72] observed no significant reduction in acetate production under heterotrophic and autotrophic growth conditions, when they knocked out the *C.* *ljungdahlii pta* gene, and additionally knocking out *adhE1* and *adhE2* actually increased ethanol formation, but only on fructose. However, the studies cannot be compared since growth conditions differed, and at least two pathways are involved in ethanol biosynthesis. Via the indirect AOR route, acetate is reduced by AOR to acetaldehyde, which is subsequently converted into ethanol by an alcohol dehydrogenase (ADH). The mRNA of AOR [73] as well as the enzymes AOR and ADH [45] were found to be highly abundant in *C.* *ljungdahlii* growing on syngas. However, it was found just recently, that ethanol and acetate formation in *C.* *ljungdahlii* is also regulated by posttranslational modification ‘protein lysine acetylation’ of AdhE1 and Pta, influencing the enzyme activities [74].

In the ultimate application of hexanol-producing *Clostridium* strains, industrial off-gases that fluctuate in gas composition and contain impurities would be used as a substrate instead of the pure synthetic gas used in this study. Microorganisms tolerate variable conditions much better than chemical catalysts [17]. *Clostridium* species have already been shown to tolerate several impurities—for example, *C.* *carboxidivorans* can tolerate up to 0.35% ethane, 1.4% ethylene and 0.1% acetylene [75], whereas *C.* *ljungdahlii* can tolerate up to 2.7% hydrogen sulfide [76] but is inhibited by 0.01% hydrogen cyanide [77]. The industrial-scale fermentation of *C.* *autoethanogenum* using steel mill gases for the production of ethanol has already been achieved by LanzaTech [78].

## Conclusions

In conclusion, we have shown that the transfer of complex metabolic pathways from *C. kluyveri* and *C. acetobutylicum* to the syngas-fermenting species *C. ljungdahlii* is a suitable approach to widen the product spectrum towards longer chain alcohols for an application as biofuels. Furthermore, we provided in vivo evidence to confirm the functions of the *C. kluyveri* genes predicted by Seedorf et al*.* [41] to cover the reactions from acetyl-CoA to butyryl-CoA and hexanoyl-CoA. For further strain development, we removed the antibiotic resistance marker using the CRISPR/Cas9 system and used the resulting marker-free strain as a new background for the introduction of an additional gene cluster from *C.* *carboxidivorans*, thus increasing hexanol production even further. We could show the usefulness and functionality of the chosen strategy for conjugation and genomic integration of large biosynthesis pathways into foreign host organisms. By applying the workflow described herein, further genes or sequences can be added, exchanged or deleted to improve the formation of non-native products by *C.* *ljungdahlii* growing on syngas as a carbon and energy source.

## Supplementary Information

**Additional file 1: Table S1**. Primer list for construction of shuttle vectors. **Table S2**. Nucleic acids of the hexanol gene cluster Hex#15. **Figure S1**. Overview on the cloning steps and vector maps of the different plasmids involved in the generation of the hexanol biosynthesis plasmid pIM Hex#15. **Table S3**. Oligo- nucleotide primers for the generation of hexanol gene construct Hex#15. **Figure S2**. Structure of the gene clusters for the biosynthesis of hexanol. **Table S4**. Oligonucleotide primers for the generation of hexanol gene construct pIM Ccar1 and pIM Ccar2. **Table S5**. Oligonucleotide primers for the generation of CRISPR plasmid pCJRK Δ*erm*C. **Table S6**. Transition list of metabolites for LC-MS/MS analysis with Q1 and Q3 masses of analyzed fragments. **Table S7**. Transition list of peptides for LC-MS/MS analysis. **Figure S3**. Maximum product concentrations and optical densities achieved by *C. lju* pIM Hex#15 after 382 h in standing serum bottle cultivations with different gas compositions as sole carbon and energy source. **Figure S4**. Schematic overview of the genomic integration locus of the butanol/hexanol construct in *C. ljungdahlii* wildtype genome (A) and verification of the correct predicted position with three different control PCRs (B). **Table S8**. Oligo nucleotide primers for the verification of Hex#15 integration locus. **Figure S5**. A) Visualization of PCR to check for the removal of the antibiotic resistance gene *ermC*. **Table S9**. Oligo nucleotide primers for the verification of *ermC* excision and loss of CRISPR plasmid. **Figure S6**. Product concentrations of cultures grown from single colonies of *C. ljungdahlii* wildtype after conjugation with plasmids for butanol/hexanol formation from *C. carboxidivorans*. **Table S10**. Enzyme content [ng_enzyme_ µg^−1^_soluble protein_] of butanol/hexanol pathway and native *C. ljungdahlii* enzymes in *C. lju* pIM Hex#15 and *C. lju* Hex#15^gInt^ in the early logarithmic (OD_600_ 0.3 – 0.5), late logarithmic (OD_600_ 0.5 – 1.0) and stationary growth phase (OD_600_ >1.0) in a 2-L fermentation with continuous gas supply with 20% CO_2_, 80% H_2_. **Table S11**. Enzyme content [ng_enzyme_ µg^-1^_soluble protein_] of butanol/hexanol pathway and native *C. ljungdahlii* enzymes in *C. lju* Hex#15^gInt^ Ccar1^gInt^ in the early logarithmic (OD_600_ 0.3 – 0.5) and late logarithmic (OD_600_ 0.5 – 1.0) growth phase in 2 L fermentations with constant pH 5.9 (I) and unregulated pH (II) with continuous gas supply with 20% CO_2_, 80% H_2_.

## Data Availability

All data generated or analyzed during this study are included in this article and its supplementary information file or are available from the corresponding author on request.
