# Supplementary material for: Metabolic engineering of Clostridium ljungdahlii for the production of hexanol and butanol from CO2 and H2
Source: Microb Cell Fact. 2022 May 14;21:85. doi: 10.1186/s12934-022-01802-8 (PMC9107641; doi:10.1186/s12934-022-01802-8)
Supplement: Supplementary file 1 — Additional file 1: Table S1. Primer list for construction of shuttle vectors. Table S2. Nucleic acids of the hexanol gene cluster Hex#15. Figure S1. Overview on the cloning steps and vector maps of the different plasmids involved in the generation of the hexanol biosynthesis plasmid pIM Hex#15. Table S3. Oligo- nucleotide primers for the generation of hexanol gene construct Hex#15. Figure S2. Structure of the gene clusters for the biosynthesis of hexanol. Table S4. Oligonucleotide primers for the generation of hexanol gene construct pIM Ccar1 and pIM Ccar2. Table S5. Oligonucleotide primers for the generation of CRISPR plasmid pCJRK ΔermC. Table S6. Transition list of metabolites for LC-MS/MS analysis with Q1 and Q3 masses of analyzed fragments. Table S7. Transition list of peptides for LC-MS/MS analysis. Figure S3. Maximum product concentrations and optical densities achieved by C. lju pIM Hex#15 after 382 h in standing serum bottle cultivations with different gas compositions as sole carbon and energy source. Figure S4. Schematic overview of the genomic integration locus of the butanol/hexanol construct in C. ljungdahlii wildtype genome (A) and verification of the correct predicted position with three different control PCRs (B). Table S8. Oligo nucleotide primers for the verification of Hex#15 integration locus. Figure S5. A) Visualization of PCR to check for the removal of the antibiotic resistance gene ermC. Table S9. Oligo nucleotide primers for the verification of ermC excision and loss of CRISPR plasmid. Figure S6. Product concentrations of cultures grown from single colonies of C. ljungdahlii wildtype after conjugation with plasmids for butanol/hexanol formation from C. carboxidivorans. Table S10. Enzyme content [ngenzyme µg−1soluble protein] of butanol/hexanol pathway and native C. ljungdahlii enzymes in C. lju pIM Hex#15 and C. lju Hex#15gInt in the early logarithmic (OD600 0.3 – 0.5), late logarithmic (OD600 0.5 – 1.0) and stationary growth phase (OD600 > [file 12934_2022_1802_MOESM1_ESM.docx]

Supporting Information

**Metabolic engineering of *Clostridium ljungdahlii* for the sustainable production of hexanol and butanol from CO_2_ and H_2_**

Ira Lauer^1,2^, Gabriele Philipps^1^ and Stefan Jennewein^1^

^1^Department for Industrial Biotechnology, Fraunhofer Institute for Molecular Biology and Applied Ecology IME, Forckenbeckstr. 6, 52074 Aachen, Germany

^2^Institute of Applied Microbiology, RWTH Aachen University, 52074 Aachen, Germany

# Detailed Experimental Procedures

## Microorganisms and microbial growth conditions

*Clostridium kluyveri* DSM 555, *Clostridium ljungdahlii* DSM 13528, *Clostridium carboxidivorans* DSM 15243 and *Clostridium acetobutylicum* DSM 792 (corresponds to strain ATCC 824) were obtained from the German Collection of Microorganisms and Cell Cultures (DSMZ). *Escherichia coli* NEB 10β cells were purchased from New England Biolabs GmbH. Stbl3 with plasmid pRK2013 served as helper strain for conjugation (GeneArt Seamless PLUS Cloning and Assembly Kit).

DSMZ medium 52 was used for cultivation of *C. kluyveri* DSM 555 and was prepared following the instructions described by the DSMZ. It contained the following ingredients per liter of distilled water: 10 g C_2_H_3_KO_2_, 0.31 g K_2_HPO_4_, 0.23 g KH_2_PO_4_, 0.25 g NH_4_Cl, 0.20 g MgSO_4_ x 7 H_2_O, 1 mL trace element SL-10 stock solution, 1 mL selenite-tungstate stock solution, 1 g yeast extract (Oxoid), 2 mg L^-1^ resazurin as redox indicator, 20 mL ethanol, 2.5g NaHCO_3_, 1 mL seven vitamin stock solution, 0.25 g cysteine-HCl x H_2_O and 0.25 g Na_2_S x 9 H_2_O. Trace element SL-10 stock solution contained per liter of distilled water: 10 mL HCl (25%, 7.7 M), 1.5 g FeCl_2_ x 4 H_2_O, 70 mg ZnCl_2_, 100 mg MnCl_2_ x 4 H_2_O, 6 mg H_3_BO_3_, 190 mg CoCl_2_ x 6 H_2_O, 2 mg CuCl_2_ x 2 H_2_O, 24 mg NiCl_2_ x 6 H_2_O, 36 mg Na_2_MoO_4_ x 2 H_2_O, Selenite and tungstate stock solution contained per liter of distilled water: 0.5 g NaOH, 3 mg Na_2_SeO_3_ x 5 H_2_O, 4 mg Na_2_WO_4_ x 2 H_2_O. Seven vitamin stock solution contained per liter of distilled water: 100 mg vitamin B_12_, 80 mg p-aminobenzoic acid, 20 mg D(+)-biotin, 200 mg nicotinic acid, 100 mg calcium pantothenate, 300 mg pyridoxine hydrochloride, 200 mg thiamine-HCl x 2 H_2_O. The final pH of the medium was adjusted to 6.8 using cysteine-HCl solution and acetic acid and the volume was adjusted to 1 L.

*C. ljungdahlii* DSM 13528 was cultivated in modified ATCC 1754 (defined) medium or YTF (complex) medium.

Modified ATCC 1794 medium was prepared according the instructions described by the ATCC with minor modifications. The medium contained the following ingredients per liter of distilled water: 1 g NH_4_Cl, 0.1 g KCl, 0.2 g MgSO_4_ x 7 H_2_O, 0.8 g NaCl, 0.1 g KH_2_PO_4_, 20 mg CaCl_2_ x 2 H_2_O, 1 g yeast extract (Oxoid), 10 mL trace elements stock solution, 10 mL Wolfe's vitamin stock solution, 10 mL reducing agent, and 2 mg resazurin as redox indicator, Trace element stock solution contained per liter of distilled water: 2 g nitrilotriacetic acid; 1 g MnSO4 x H_2_O, 0.8 g Fe(SO_4_)_2_(NH_4_)_2_ x 6 H_2_O, 0.2 g CoCl_2_ x 6 H_2_O, 0.2 mg ZnSO_4_ x 7 H_2_O, 20 mg CuCl_2_ x 2 H_2_O, 20 mg NiCl_2_ x 6 H_2_O, 20 mg Na_2_MoO_4_ x 2 H_2_O, 20 mg Na_2_SeO_4_, 20 mg Na_2_WO_4_. Wolfe’s vitamin stock solution contained per liter of distilled water: 2.0 mg Biotin, 2.0 mg Folic acid, 10.0 mg Pyridoxine hydrochloride, 5.0 mg Thiamine HCl, 5.0 mg Riboflavin, 5.0 mg Nicotinic acid, 5.0 mg Calcium D-(+)-pantothenate, 0.1 mg Vitamin B12, 5.0 mg p-Aminobenzoic acid, 5.0 mg Thioctic acid. L-cysteine stock solution with 75 g L^-1^ was used as the only reducing agent. 20 mM or 100 mM BisTris served as buffering agent instead of NaHCO_3_ and the final pH was set to 6.0. For heterotrophic growth 20 mL of 25% (w/v) sterile filtered fructose solution was used.

Cultivation of pre-cultures was performed in a Bactron 600 (Shellab) anaerobic workbench or in a Whitley A35 anaerobic chamber (Don Whitley Scientific) with an anaerobic atmosphere consisting of 10% carbon dioxide, 5 % hydrogen and 85 % nitrogen. Fermentation was performed as described in the results and discussion section. *E. coli* NEB 10β cells were plated on LB agar after transformation. Liquid cultivation of *E. coli* strains was performed in LB medium. For selection antibiotics were added in the following concentrations (f.c.): 100 mg L^-1^ ampicillin, 100 mg L^-1^ erythromycin, and 3.4 mg L^-1^ chloramphenicol for *E. coli* NEB 10β when performing triple selection and 100 mg/L kanamycin for *E. coli* Stbl3 with pRK2013. Unless stated otherwise, 200 mg L^‑1^ d-cycloserine, 4 mg L^-1^ clarithromycin and/or 4 mg L^-1^ thiamphenicol were added for selection of *C. ljungdahlii* mutant strains. When strains were cultivated on solid plate, 1.5% (w/v) BD Difco Agar (Becton-Dickinson, Franklin Lakes, NJ, USA) was added to the medium.

## Generation of the *E. coli* – *Clostridium* shuttle vectors

Restriction enzymes were purchased from New England Biolabs and used according to the manufacturers recommendations. All PCR amplifications were performed with Phusion High Fidelity DNA Polymerase (New England Biolabs) according to the manufacturer instructions unless stated otherwise. One-step isothermal DNA assembly was performed according to Gibson et al. (2009). A total amount of 100 ng DNA (vector backbone and insert(s) in equimolar concentrations) in a volume of 5 µL was mixed with 15 µL of the assembly master mix. Reactions were incubated at 50°C for 60 minutes. Aliquots of the assembly reaction were used for transformation of *E. coli* NEB 10β cells

### pIM-SLIC

The vector sequence of the shuttle vector pIM-SLIC (Phillips *et al.*, 2015) is derived from pIM-traJ. To support sequence and ligation independent cloning (SLIC) and Gibson assembly of heterologous gene clusters into pIM-traJ a multiple cloning site containing fragment of pET41a was inserted downstream of the vector encoded *ptb* promoter. The fragment was PCR-amplified using primers SLIC-site-SacII for and SLIC-site-SacII rev (Table S 1). The PCR product was inserted via *Sac*II restriction site into pIM-traJ to generate pIM-SLIC (Figure S 1).

### pIM-SLIC-Dest

For generation of pIM-SLIC-Dest (Figure S 1) the Gateway conversion kit (Life Technologies) was employed according to the manufacturer recommendations. The Gateway cassette template containing a chloramphenicol acetyl transferase (Cm^R^) and a CcdB gene was amplified using primers GW-pIM-SLIC for and GW-pIM-SLIC rev (Table S 1) and was inserted via isothermal assembly into the pIM-SLIC vector.

### pIM-SLIC-H1C9

pIM-SLIC-traJ-H1C9 (Figure S 1) was generated based on pIM-SLIC by Quick change using Phusion High Fidelity DNA Polymerase with primers QC_himar_for1 and QC_himar_rev1(Table S 1) to exchange two amino acids from the *Himar1* transposase to the hyperactive variant H1 C9 (Lampe *et al.*, 1999).

### pIM-SLIC-repH-H1C9

pIM-SLIC-repH-H1C9 (Figure S 1) was generated based on pIM-SLIC-H1C9 by PCR and Gibson assembly using primers pIM-repH SLIC for1 and pIM-repH SLIC rev2 for amplification of the origin of replication *repH* and primers pIM-repH SLICrev1 and pIM-repH SLIC for2 (Table S 1) for amplification of the vector backbone with Phusion High Fidelity DNA Polymerase. After size separation on an agarose gel the corresponding bands were cut from the gel and purified with the NucleoSpin Gel and PCR clean-up kit from Macherey and Nagel. After DpnI-digest (NEB) a Gibson assembly reaction was performed according to (Gibson *et al.*, 2009). 5 µL were used for transformation of chemically competent *E.coli* NEB 10β cells.

Table S 1. Primer list for construction of shuttle vectors

| Primer Name | Primer sequence 5’ → 3’ |
| --- | --- |
| SLIC-site-SacII for 1 | CGTACCCCGCGGGGCTTTGTTTAGCAGCCTAGGTATTAATCAATTAG |
| SLIC-site-SacII rev | CGTACCCCGCGGGTACCGGTGGTGGCTCCGGTGATGACGACGACAAG |
| GW-pIM-SLIC for 1 | GTACCGGTGGTGGCTCCGGTGATGACGACGACAAGATCACAAGTTTGTACAAAAAAGCTG |
| GW-pIM-SLIC rev | GGCTTTGTTTAGCAGCCTAGGTATTAATCAATTAGATCACCACTTTGTACAAGAAAGC |
| QC_himar_for1 | ACAA**AG**ACGTGTTGATGATTCT**A**AGCGGTGTTTGCAGCTGTTAACTC |
| QC_himar_rev1 | CACCGCT**T**AGAATCATCAACACGT**CT**TTGTTTTTGGTCAAATGTGAG |
| pIM-repH SLIC for1 | CTGGCGCGCCGCCATTATTTTTTTGAACAATTGACAATTCATTTCTTATTTTTTATTAAGTGATAG |
| pIM-repH SLIC rev2 | TTGCCCACTGGCCGGCCGCTTATAATCCATAACAATCATCCTTTCTGTGACACTGTCAGACAC |
| pIM-repH SLIC for2 | GATGATTGTTATGGATTATAAGCGGCCGGCCAGTGGGCAAGTTGAAAAATTCACAAAAATGTGG |
| pIM-repH SLIC rev1 | GAAATGAATTGTCAATTGTTCAAAAAAATAATGGCGGCGCGCCAGAGCCTACGAGTTCCGAACTAG |

## Generation of the hexanol production cluster Hex#15

The biosynthesis of hexanol from acetyl-CoA requires ten reaction steps involving 13 enzymes. The corresponding genes were selected from *C. kluyveri* (DSM 555) and *C. acetobutylicum* (ATCC 824). Promoter sequences were amplified from genomic DNA from *C. ljungdahlii* DSM 13528. The employed nucleic acids are listed in Table S 2. *C. kluyveri* genomic DNA was sourced from the German Collection of Microorganisms and Cell Cultures (DSMZ). Genomic DNA from *C. acetobutylicum* and *C. ljungdahlii* was prepared with the NucloSpin Tissue kit (Macherey Nagel) from actively growing cells according to the manufacturer’s instructions.

Table S 2: Nucleic acids of the hexanol gene cluster Hex#15

| Name | Source | Accession number | Orientation | Genomic region (bp) | Size (bp) |
| --- | --- | --- | --- | --- | --- |
| Thiolase 1 (ThlA1) | *C. kluyveri* | CP000673.1 | c | 3734524 - 3735705 | 1182 |
| Crotonase 1 (Crt1) | *C. kluyveri* | CP000673.1 |  | 433169 - 433948 | 780 |
| Butyryl-CoA dehydrogenase (Bcd1) | *C. kluyveri* | CP000673.1 |  | 434124 - 435263 | 1140 |
| Electron transferring protein B1 (EtfB1) | *C. kluyveri* | CP000673.1 |  | 435280 - 436059 | 780 |
| Electron transferring protein A1 (EtfA1) | *C. kluyveri* | CP000673.1 |  | 436079 - 437083 | 1005 |
| Hydroxybutyryl-CoA-Dehydrogenase (Hbd 1) | *C. kluyveri* | CP000673.1 |  | 437268 - 438116 | 849 |
| CO dehydrogenase promoter (COdh promoter) | *C. ljungdahlii* | CP001666.1 | c | 4093356 - 4093898 | 543 |
| Bifunctional aldehyde/alcohol dehydrogenase E2 (AdhE2) | *C. acetobutylicum* | NG_035407.1 | c | 344 - 2920 | 2577 |
| Phosphate acetyltransferase – Acetate kinase (Pta-Ack promoter) | *C. ljungdahlii* | CP001666.1 |  | 1376039 - 1376300 | 287 |
| Thiolase 2 (ThlA 2) | *C. kluyveri* | CP000673.1 | c | 3736586 - 3737761 | 1176 |
| Crotonase 2 (Crt 2) | *C. kluyveri* | CP000673.1 |  | 2603684 - 2604457 | 774 |
| Butyryl-CoA dehydrogenase 2 (Bcd 2) | *C. kluyveri* | CP000673.1 |  | 617473 - 619161 | 1689 |
| Electron transferring proteins A2 (EtfA2) | *C. kluyveri* | CP000673.1 | c | 3563939 - 3564937 | 999 |
| Electron transferring proteins B2 (EtfB2) | *C. kluyveri* | CP000673.1 | c | 3564940 - 3565743 | 804 |
| Hydroxybutyryl-CoA-Dehydrogenase 2 (Hbd 2) | *C. kluyveri* | CP000673.1 | c | 2868731 - 2869690 | 960 |

c – complementary

The complete hexanol biosynthesis cluster was assembled from 10 individually PCR-amplified DNA fragments in a multi-step process employing different molecular biological methods: (a) PCR amplification of individual fragments, (b) SOE-PCR to combine fragments to three sub-cluster units (c) Cloning of sub-cluster units into pDONR vectors (Life Technologies) suitable for multisite gateway cloning with three pENTR vectors and (d) Assembly of sub-cluster units from pENTR vectors in a Multisite gateway reaction into the destination vector pIM-SLIC-Dest. In a later step the origin of replication was exchanged from *repL* to *repH* resulting in the final vector pIM-Hex#15. The different plasmids involved in the cloning steps are depicted in Figure S 1. The detailed cloning steps are described in the following section.

Figure S 1: Overview on the cloning steps and vector maps of different plasmids involved in the generation of the hexanol biosynthesis plasmid pIM Hex#15.

### pENTR-1-4-C4

The first part of the hexanol cluster was assembled with the generation of the entry vector pENTR-1-4-C4. Therefore, the Thiolase 1 (*thl1*) gene and a 5kb-fragment containing genes *crt1*, *bcd1*, *etfB1*, *etfA1* and *hbd1* were amplified with Phusion DNA polymerase (NEB) using primers listed in Table S 3. Fragments were purified, assembled by SOE-PCR and cloned in a BP-reaction into the appropriate pDONR vector (Figure S 1).

### pENTR-4r3r-adhE2

For generation of the entry vector pENTR-4r3r-adhE2 a native promoter region derived from the sequence upstream of the genes *pta-ack* was amplified using primers listed in Table S 3 with *C. ljungdahlii* genomic DNA serving as template. This promotor is known to be constitutively active in heterotrophic and autotrophic grown cultures (Bengelsdorf *et al.*, 2016). The bifunctional alcohol dehydrogenase was amplified from *C. acetobutylicum* genomic DNA (primers listed in Table S 3). This gene was chosen since it is known to efficiently process the last reduction step from the butyryl-CoA ester towards the primary alcohol butanol and we assumed a similar reactivity for the reduction from crotonyl-CoA to hexanol. The fragments were assembled in an SOE-PCR reaction and cloned into the suitable pDONR vector (Figure S 1).

### pENTR-3-2-C6

For generation of the entry vector pENTR-3-2-C6, a native promoter region from the CO dehydrogenase gene derived from the genomic DNA of *C. ljungdahlii* was amplified. This promotor is known to be constitutively active in cultures grown under heterotrophic and autotrophic conditions. Five further PCR-fragments were generated with the genes 1) *thlA2*, 2) *crt2*, 3) *bcd2*, 4) *etfB2* and *etfA2*, and 5) *hbd2* using genomic DNA from *C. kluyveri* as a template. The used primers created overlaps between the fragments necessary for assembling the fragment in a splicing by overlap extension (SOE)-PCR. All primers are listed in Table S 3. Applied conditions for the SOE-PCR were adapted from (Shevchuk *et al.*, 2004). Two gel-purified DNA fragments each were assembled in separate reactions: (a) codH-promoter + ThlA2, (b) Crt2 + Bcd2 and (c) EtfB2/A2 + Hbd2. DNA fragments were mixed in equimolar concentrations with a total of 1 µg DNA per reaction (660 fmol DNA fragment, 1x Phusion HF-buffer, 0.25 mM dNTPs, 2 Units Phusion HF DNA polymerase). After an initial denaturation (98°C 2min) a PCR cycle (98°C 10s, 60°C 20s) was run 12 times with a final elongation step (72°C 2min). Assembly products were purified with a PCR-purification kit (Gel extraction and PCR-purification kit, Macherey Nagel). The joining reaction of the 3 assembly products was performed with a total of 1.5 µg DNA as described for the assembly products. The reaction was supplemented after these 12 cycles with 100 µL PCR mix including primers attB3-WL COdh for and attB2-CKLU-Hbd2 rev (final concentration of 0.25 µM each). After an initial denaturation (98°C 2min) a PCR cycle (98°C 10s, 58°C 20s, 72°C 5min) was run 20 times with a final elongation step (72°C 5min). The PCR reaction was purified with a PCR-purification kit. The eluate of the PCR-purification was subjected to a BP-reaction into pDONR-vector with attachment sites attB3-attB2 according to the manufacturer recommendations (Figure S 1).

Table S 3: Oligo nucleotide primers for the generation of hexanol gene construct Hex#15

| PCR fragment | Primer name | Primer sequence 5’ → 3’ |
| --- | --- | --- |
| thlA1 | attB1-rbs-CKLU-ThlA1 for | GGGGACAAGTTTGTACAAAAAAGCAGGCTTAGGAGGATTTGTTATGAGAGAAGTAGTTATTGTAAGTGC |
|  | CKLU_ThlA1-crt1 rev | CAAGAATGATATTTTTAAATTCCATATTACTCCTCCCTCCTAAACGTTTAAATTATCTTTCAACTACTACAGCGGTTCCTTGTCC |
| crt1 - hbd1 | CLKU_Crt1-ThlA1 for | GTAGTTGAAAGATAATTTAAACGTTTAGGAGGGAGGAGTAATATGGAATTTAAAAATATCATTCTTGAAAAGGATGGAAATGTGG |
|  | attB4-CKLU-Hbd1 rev | GGGGACAACTTTGTATAGAAAAGTTGGGTGCTATCTAAATCACATAATTTTAATAAGCGAAGAATCCTTTTCCTGATTTTCTTCCAAGC |
| pta-ack promoter from *C. ljungdahlii* | attB4r-Pta-P ext for | GGGGACAACTTTTCTATACAAAGTTGCCTAAGTGAAATATATACATATTATAACAATAAAATAAG |
|  | PtaP-CACE-AdhE2 SLIC rev | GATTTGTAACTTTCATTTATATACACTCCTTTATAAAATTTATTATTAGTTTAAATTTAACACAAAATTACACACACTTATAC |
| AdhE2 | CACE-AdhE2-PtaP SLIC for | GTTAAATTTAAACTAATAATAAATTTTATAAAGGAGTGTATATAAATGAAAGTTACAAATCAAAAAGAACTAAAACAAAAGCTAAATG |
|  | attB3r-CACE-AdhE2 rev | GGGGACAACTTTATTATACAAAGTTGTTTAAAATGATTTTATATAGATATCCTTAAGTTCACTTATAAGTGGATAC |
| codH promoter from *C. ljungdahlii* | attB3-WL COdh for | GGGGACAACTTTGTATAATAAAGTTGAGATAGTCATAATAGTTCCAGAATAGTTTAATTTAG |
|  | WL-Codh-P-CKLU-ThlA2 rev | GCACTTACAATAACTGCATCTTTCATAAAATGAACCCTCCTAATAAAGAAATAACTTCAATTTTTGTTGTAATTAC |
| ThlA 2 | CKLU-ThlA2-Codh-P for | TTAGGAGGGTTCATTTTATGAAAGATGCAGTTATTGTAAGTGCAGTAAGAACAGC |
|  | CKLU-ThlA2-Crt2 rev | GTTTTATACGTCATTATAAAGCCTCCTGCCTTAGCCATTATATAAAATTATCTTTCAACTATTAGTGCAGTTCCCATTCCTC |
| Crt 2 | CKLU-Crt2-ThlA2 for | GATAATTTTATATAATGGCTAAGGCAGGAGGCTTTATAATGACGTATAAAACTTTATTATTAGAGAAGCAGAATGGG |
|  | CKLU-Crt2-Bcd2 rev | CATAAGACCATCTCCCTAAATATAATTTTCAACCGAATTGAAGTTACTTAAAGACTGGCTTTCTTTTTTCAATAAATGCAGC |
| Bcd 2 | CKLU-Bcd2-Crt2 for | GTAACTTCAATTCGGTTGAAAATTATATTTAGGGAGATGGTCTTATGAACTTTGAACTCACCAAAGAACAACAAATGATTAGAGATAATG |
|  | CKLU-Bcd2-etfB2 rev | GCTTCTTTCCTTTCTCTCAAACCTCTTAAAAAATTACAAATTCTATTTTAAAGCCTCAATAAGCAGCGGCACAATTTC |
| EtfB 2 - EtfA 2 | CKLU-etfB2-Bcd2 for | GTAATTTTTTAAGAGGTTTGAGAGAAAGGAAAGAAGCTTTAATGGATATTATAGTTTTAGTTAAACAAGTTCCGGATATGGAAAAAG |
|  | CKLU-etfA2-Hbd2 rev | CATCCTCCTGAATTCTATATTTATAGCCCTTCTTCTTTATTATTTAACAATTTATAGTTTCTAAACTGCTTTCTTTTATCTTAG |
| Hbd 2 | CKLU-Hbd2-etfA2 for | AATAAAGAAGAAGGGCTATAAATATAGAATTCAGGAGGATGTTATGGATATAAAAAATGTAGCTGTACTTGGTACTGGTAC |
|  | attB2-CKLU-Hbd2 rev | GGGGACCACTTTGTACAAGAAAGCTGGGTTTTATATATTCCTTTCAATGGATTTATCATTTTTATCTTTTTCCAAAAAATCC |
| ThlA1-Hbd2 | ThlA1-CKLU-thlRBS-pSLIC-repH for | GTACCGGTGGTGGCTCCGGTGATGACGACGACAAGAGTCCCATGAGGAGGATTTGTTATGAGAGAAGTAGTTATTGTAAGTGC |
|  | Adh-pSLIC-repH rev | GGTATTAATCAATTAGTGGTGGTGGTGGTGGTGGTGGTGCTCGAGTGCggccTTATATATTCCTTTCAATGGATTTATCATTTTTATCTTTTTCC |

Figure S 2: Structure of the gene clusters for the biosynthesis of hexanol. *thlA1/2*: thiolase A1/2, *crt1/2*: crotonase 1/2, *bcd1/2*: butyryl-CoA-dehydrogenase 1/2, *etfA1/2*: electron transferring protein A1/2, *etfB1/2*: electron transferring protein B1/2, *hbd1/2*: hydroxybutyryl-CoA dehydrogenase 1/2 (all genes from *C. kluyveri*); *adhE2*: bifunctional aldehyde-alcohol-dehydrogenase from *C. acetobutylicum*, *ptb*: phosphate butyryltransferase, *pta-ack*: phosphate acetyltransferase – acetate kinase, *codH*: carbon monoxide dehydrogenase. Coloured straight arrows represent individual genes. Genes that are organized in gene clusters in *C. kluyveri* genome are shown in gray boxes. Curved arrows represent promoter regions from *C. acetobutylicum* (*ptb*-promoter) and *C. ljungdahlii* (*pta-ack*- promoter and *codH*-promoter). The *ptb*-promoter coloured in gray is not present in the pENTR-1-4-C4 vector but is provided by the pIM SLIC plasmid.

### pIM Hex#12

The three generated entry vectors were assembled to the complete hexanol biosynthesis gene cluster in a multisite gateway reaction (Life Technologies). The employed destination vector pIM-SLIC-Dest (10fmol per reaction) was incubated with 20 fmol of each of the three pENTR vectors containing the sub-cluster units with LR clonase enzyme mix according to the manufacturer recommendations at room temperature overnight. Positive clones were identified by restriction digest and verified by sequencing. The final working construct was designated pIM-Hex12 (Figure S 1).

### pIM Hex#15

As we did not obtain colonies after conjugation of *C. ljungdahlii* with *E. coli* harboring the pIM Hex#12, we decided to exchange the gram positive origin of replication *repL* to the *repH*, which had been shown to result in a higher number of transconjugants in previous experiments (Philipps *et al.*, 2019). The assembled hexanol gene cluster from pIM Hex12 was amplified with primers (ThlA1-CKLU-thlRBS-pSLIC-repH for and Adh-pSLIC-repH rev ) and inserted into the NotI-NcoI-linearized vector pIM-SLIC-H1C9-repH in an isothermal assembly (Gibson *et al.*, 2009) resulting in pIM Hex#15 (Figure S 1).

## Generation of the hexanol production clusters Ccar1 and Ccar2

The genes used for cloning of the hexanol production clusters Ccar1 and Ccar2 are organized as operons in the genome of *C. carboxidivorans* (Accession number CP011803). The operons Ccar1 (Ccar_RS22775 – Ccar_RS22800: *crt*, *hbd*, *thl*, *bcd*, *etfB*, *etfA*) and Ccar2 (Ccar_RS01400 – Ccar_RS01430: *crt*, *hbd*, *thl*, *ech*, *bcd*, *etfB*, *etfA*) were amplified from the genome of *C. carboxidivorans* (Table S 4). The *thlA* promoter was amplified from the genome of *C. acetobutylicum* and the *codH* promoter from the genome of *C. ljungdahlii* (Table S 4). The vector backbone pIM-SLIC-H1C9-repH was amplified from pIM Hex#15 (Table S 4). Vector backbone, *thlA* promoter from *C. acetobutylicum* and Ccar1 as well as vector backbone, *codH* promoter from *C. ljungdahlii* and Ccar2 were assembled in an isothermal assembly (Gibson *et al.*, 2009) resulting in pIM Ccar1 and pIM Ccar2, respectively.

Table S 4: Oligo nucleotide primers for the generation of hexanol gene construct pIM Ccar1 and pIM Ccar2

| PCR fragment | Primer name | Primer sequence 5’ → 3’ |
| --- | --- | --- |
| Ccar1 | Cluster1_Ccar_thl_SLIC_for01 | CCGTATCAAAATTTAGGAGGTTAGTTAGTTGGAATATAAAAATATTAAAGTGGAAAAGGAAAATC |
|  | Cluster1P_Ccar_SLIC_rev02 | CCTCCGCGGCATAAAGTAGTGCAACCTCCTACCCCTCATATATTGATAAAAAATATAAATAATTG |
| *thlA* promoter from *C. acetobutylicum* | Thl_Cluster1_Ccar_SLIC_rev01 | CCTTTTCCACTTTAATATTTTTATATTCCAACTAACTAACCTCCTAAATTTTGATACGGGGTAAC |
|  | Thl_Bb_SLIC_for01 | CTGGCAAATAGTGACTTCTGAAATGAGCTTTTTAACAAAATATATTGATAAAAATAATAATAGTG |
| backbone for pIM Ccar1 | Bb_thl_SLIC_rev01 | TATTATTATTTTTATCAATATATTTTGTTAAAAAGCTCATTTCAGAAGTCACTATTTGCCAGAAC |
|  | Cluster1P_Ccar_SLIC_for02 | AATTATTTATATTTTTTATCAATATATGAGGGGTAGGAGGTTGCACTACTTTATGCCGCGGAGGC |
| Ccar2 | Cluster2_Ccar_cod_SLIC_for01 | AGTTATTTCTTTAAGGAGGGAATTATTAAAATGGGGTATGAAAACGTTGTTTTAGAGAAACAAGG |
|  | Cluster2P_Ccar_SLIC_rev01 | GGATTTAAATCGGCCGCGCCGGCCTCCGGTCATCTTAGAAAAATATCATCTAAAATAAAAATTTC |
| *codH* promoter from *C. ljungdahlii* | cod_Cluster2_Ccar_SLIC_rev01 | TTGTTTCTCTAAAACAACGTTTTCATACCCCATTTTAATAATTCCCTCCTTAAAGAAATAACTTC |
|  | cod_Bb_Ccar_SLIC_for01 | TAACAAAATATATTGATAAAAATAATAATACCAAAATATGTCACACGCAATTGCATATTTCAAAC |
| backbone for pIM Ccar2 | Bb_thl_SLIC_rev01 | TATTATTATTTTTATCAATATATTTTGTTAAAAAGCTCATTTCAGAAGTCACTATTTGCCAGAAC |
|  | Bb_Cluster2P_Ccar_SLIC_for01 | GAAATTTTTATTTTAGATGATATTTTTCTAAGATGACCGGAGGCCGGCGCGGCCGATTTAAATCC |

## Generation of the CRISPR/Cas9 plasmid for excision of *ermC* gene

The basic CRISPR/Cas9 plasmid was designed by Christian Janke and generated by Richard Kosinski (Industrial Biotechnology, Fraunhofer IME Aachen) containing the origin of replication *repH* (pCB102) for Clostridium and the origin of replication pMB1 from pUC19 for *E. coli*, *cas9* under control of the lactose inducible promoter *bgaR-*P_Lac_, sgRNA under control of the *araE* promoter from *C. acetobutylicum* as well as *catP* and *bla* genes as selection markers on a pUC19 backbone. The CRISPR/Cas9 plasmid specific for *ermC* excision was generated using the primers listed in Table S 5. The N20 sequence for target site recognition was designed using CRISPRdirect (Naito *et al.*, 2015) and introduced via overlapping primers.

Table S 5: Oligo nucleotide primers for the generation of CRISPR plasmid pCJRK Δ*ermC*.

| Primer Name | Primer sequence 5’ → 3’ |
| --- | --- |
| N20_mlsR_1_for | TTTCGTCGACATAAGTGAGCTATTCACTTTGTTTTAGAGCTAGAAATAGCAAGTTAAAATAAG |
| N20_mlsR_1_rev | GCTCTAAAACAAAGTGAATAGCTCACTTATGTCGACGAAAACTCCTCCTTAAGATTTATATATG |
| bla_SLIC_for | GGAACCGGAGCTGAATGAAGCCATACCAAACGACGAG |
| bla_SLIC_rev | CGTTTGGTATGGCTTCATTCAGCTCCGGTTCCCAACG |
| Crispr mlsR_VF1_for | TCGGTACCCGGGGATCCTCTAGAGTCGACCTGCAG |
| Crispr mlsR_VF2_rev | ATGGCGCCTGATGCGGTATTTTCTCCTTAC |
| Crispr mlsR hex_VF2_HA1_for | AGATGCGTAAGGAGAAAATACCGCATCAGGCGCCATGCTGTTATAAAAATAAGAAGCCTG |
| Crispr mlsR hex_HA1_HA2_for | ATATGCTCAATCCAATCCATAGTTATCCAATTAAGAATAGCAGCATGACAAACTGTATAG |
| Crispr mlsR hex_HA1_HA2_rev | TACAGTTTGTCATGCTGCTATTCTTAATTGGATAACTATGGATTGGATTGAGCATATAGG |
| Crispr mlsR hex_VF1_HA2_rev2 | ACTCTAGAGGATCCCCGGGTACCGAGTAGACCGGGGACTTATCAGCCAACCTGTTAAAGG |

## Conjugation (triparental mating) in *C. ljungdahlii*

The conjugation was performed as described by Philipps *et al.* (2019). The *E. coli* donor strain NEB10β with the respective plasmid of interest and the *E. coli* helper strain Stbl3 harboring the helper plasmid pRK2013 necessary to allow DNA transfer by conjugation were grown in LB medium (40 mL and 10 mL respectively) in the presence of appropriate antibiotics until an OD_600_ of 0.2 - 0.5 was reached. Furthermore, the recipient strain *C. ljungdahlii* was grown to an OD_600_ of 0.2 -0.3 in YTF medium. After centrifugation and washing of the cells they were mixed, spread onto YTF Agar plates without antibiotics and incubated anaerobically at 37 °C. After 12 – 16 h the cells were washed from the plates and poured into a Petri dish. 25 mL of YTF medium containing 1.5 % low melt agarose with 200 mg L^-1^ D-cycloserine, 2 mg L^-1^ clarithromycin and 2 mg L^-1^ thiamphenicol added for selection for the plasmid were mixed with the culture. After solidification at room temperature for 1 h, the plates were incubated anaerobically at 37 °C. When conjugation was performed in Whitley A35 anaerobic chamber (Don Whitley Scientific Ltd., Shipley, United Kingdom), cells were poured into difco agar (Becton Dickinson GmbH, Heidelberg, Germany) and incubated at 34 °C. Single colonies were picked and used for inoculation of 5 mL YTF medium (containing 200 mg/L D-cycloserine, 4 mg/L clarithromycin, 4 mg/L thiamphenicol). After outgrowth, an aliquot of the cell suspension was plated again on YTF plates with antibiotics for retrieval of single colonies to separate the cells from *E. coli* donor cells potentially adhering to the initially picked colonies. *C. ljungdahlii* transconjugant strains were analyzed for the presence of pIM Hex#15 or pIM Ccar1/pIM Ccar2.

.

## Intracellular metabolite analysis by LC-MSMS

Intracellular metabolites were analyzed as described in Gaida *et al.* (2016) with minor modifications. After resuspension in quenching solution, cells were lysed using a bead beater three times for 30 s at 4°C with placement on ice in between. Standard curves were prepared for the different intermediates and octanoyl-CoA served as internal standard. The transition list of analyzed metabolites is given in Table S 6.

Table S 6: Transition list of metabolites for LC-MS/MS analysis with Q1 and Q3 masses of analyzed fragments.

| Q1 Mass (Da) | Q3 Mass (Da) | Dwell Time (ms) | ID | DP (volts) | EP (volts) | CEP (volts) | CE (volts) | CXP (volts) |
| --- | --- | --- | --- | --- | --- | --- | --- | --- |
| 807.962 | 408 | 10 | Acetyl-CoA Li-salt negative | -90 | -9 | -38 | -48 | -4 |
| 807.962 | 461 | 10 | Acetyl-CoA Li-salt negative | -90 | -9 | -38 | -48 | -4 |
| 807.962 | 425.9 | 10 | Acetyl-CoA Li-salt negative | -90 | -9 | -38 | -46 | -4 |
| 835.962 | 408 | 10 | Butyryl-CoA Li-salt negative | -95 | -9.5 | -42 | -52 | -4 |
| 835.962 | 489 | 10 | Butyryl-CoA Li-salt negative | -95 | -9.5 | -42 | -48 | -4 |
| 835.962 | 425.9 | 10 | Butyryl-CoA Li-salt negative | -95 | -9.5 | -42 | -48 | -4 |
| 863.997 | 408 | 10 | Hexanoyl-CoA triLi-salt negative | -110 | -9.5 | -40 | -52 | -4 |
| 863.997 | 426 | 10 | Hexanoyl-CoA triLi-salt negative | -110 | -9.5 | -40 | -48 | -4 |
| 863.997 | 517.2 | 10 | Hexanoyl-CoA triLi-salt negative | -110 | -9.5 | -40 | -52 | -4 |
| 891.97 | 408 | 10 | Octanoyl-CoA Li-salt negative | -90 | -10 | -42 | -54 | -4 |
| 891.97 | 425.9 | 10 | Octanoyl-CoA Li-salt negative | -90 | -10 | -42 | -48 | -4 |
| 891.97 | 545.2 | 10 | Octanoyl-CoA Li-salt negative | -90 | -10 | -42 | -54 | -6 |
| 850.1 | 408.1 | 10 | Acetoacetyl-CoA sodium salt | -75 | -9.5 | -43.325 | -52 | -4 |
| 850.1 | 765.9 | 10 | Acetoacetyl-CoA sodium salt | -75 | -9.5 | -43.325 | -52 | -6 |
| 850.1 | 418.9 | 10 | Acetoacetyl-CoA sodium salt | -75 | -9.5 | -43.325 | -56 | -4 |
| 852.1 | 426 | 10 | 3-hydroxybutyryl-CoA | -90 | -9.5 | -43.397 | -48 | -4 |
| 852.1 | 408 | 10 | 3-hydroxybutyryl-CoA | -90 | -9.5 | -43.397 | -50 | -4 |
| 852.1 | 505 | 10 | 3-hydroxybutyryl-CoA | -90 | -9.5 | -43.397 | -50 | -4 |
| 834.1 | 426 | 10 | Crotonyl-CoA | -90 | -9.5 | -42.751 | -48 | -4 |
| 834.1 | 408 | 10 | Crotonyl-CoA | -90 | -9.5 | -42.751 | -50 | -4 |
| 834.1 | 487 | 10 | Crotonyl-CoA | -90 | -9.5 | -42.751 | -52 | -4 |
| 878.1 | 426 | 10 | 3-ketohexanoyl-CoA | -95 | -9.5 | -44.329 | -48 | -4 |
| 878.1 | 408 | 10 | 3-ketohexanoyl-CoA | -95 | -9.5 | -44.329 | -52 | -4 |
| 878.1 | 531 | 10 | 3-ketohexanoyl-CoA | -95 | -9.5 | -44.329 | -52 | -4 |
| 880.1 | 408 | 10 | 3-hydroxyhexanoyl-CoA | -100 | -9.5 | -44.401 | -52 | -4 |
| 880.1 | 426 | 10 | 3-hydroxyhexanoyl-CoA | -100 | -9.5 | -44.401 | -50 | -4 |
| 880.1 | 533 | 10 | 3-hydroxyhexanoyl-CoA | -100 | -9.5 | -44.401 | -52 | -4 |
| 862.1 | 408 | 10 | hexenoyl-CoA | -100 | -9.5 | -43.755 | -52 | -4 |
| 862.1 | 426 | 10 | hexenoyl-CoA | -100 | -9.5 | -43.755 | -48 | -4 |
| 862.1 | 515 | 10 | hexenoyl-CoA | -100 | -9.5 | -43.755 | -52 | -4 |

dwell time, declustering potential (DP), entrance potential (EP), colli­sion energy (CE), collision exit potential (CXP)

## Targeted Proteomics analysis by LC-MSMS

For each specific peptide sequence, we ordered a peptide version labelled with heavy isotopes ^15^N or ^13^C at the C-terminal Arginine or Lysine (SpikeTides_L, JPT Peptide Technologies GmbH, Berlin, Germany) for correct identification and a quantified peptide version (SpikeTides_TQ, JPT Peptide Technologies GmbH, Berlin, Germany) for quantification. Calibration curves for each peptide were prepared with SpikeTides_TQ to allow quantification of the peptides, which can be calculated into the amount of enzyme per soluble protein [ng µg^-1^].The transition list of measured peptides is given in Table S 7.

Table S 7: Transition list of peptides for LC-MS/MS analysis.

| Q1 [m/z[ (Da) | Q3 [m/z] (Da) | Dwell Time (ms) | ID | DP (volts) | EP (volts) | CE (volts) | CXP (volts) |
| --- | --- | --- | --- | --- | --- | --- | --- |
| 446.7480 | 674.3832 | 10 | Cklu_ThlA1 - FALASQQK | 63.7 | 10.0 | 24.9 | 12.0 |
| 412.2316 | 468.2453 | 10 | Cklu_Crt1 - ELIYTGK | 61.2 | 10.0 | 23.7 | 12.0 |
| 402.2549 | 527.3552 | 10 | Cklu_Bcd1 - YLVPLAK | 60.4 | 10.0 | 23.3 | 12.0 |
| 655.8406 | 954.5043 | 10 | Cklu_EtfB1 - NLEYDLIFAGR | 78.9 | 10.0 | 32.5 | 12.0 |
| 464.7893 | 686.4447 | 10 | Cklu_EtfA1 - IEVLEVVK | 65.0 | 10.0 | 25.6 | 12.0 |
| 371.7447 | 530.3297 | 10 | Cklu_Hbd1 - LVEIIR | 58.2 | 10.0 | 22.2 | 12.0 |
| 522.8186 | 660.3927 | 10 | Cace_AdhE - VTALIEAISK | 69.2 | 10.0 | 27.7 | 12.0 |
| 484.8106 | 726.4872 | 10 | Cklu_ThlA2 - ELGIKPLAK | 66.5 | 10.0 | 26.3 | 12.0 |
| 458.2791 | 717.4141 | 10 | Cklu_Crt2 - VVILTGEGK | 64.5 | 10.0 | 25.4 | 12.0 |
| 426.2350 | 589.3304 | 10 | Cklu_Bcd2 - YVGISGQK | 62.2 | 10.0 | 24.2 | 12.0 |
| 358.2393 | 489.3031 | 10 | Cklu_EtfB2 - ILASAIK | 57.2 | 10.0 | 21.8 | 12.0 |
| 422.7424 | 602.3508 | 10 | Cklu_EtfA2 - LENELVK | 61.9 | 10.0 | 24.1 | 12.0 |
| 413.7791 | 585.4083 | 10 | Cklu_Hbd2 - LQLALLR | 61.3 | 10.0 | 23.8 | 12.0 |
| 568.8268 | 676.4140 | 10 | Ccar_Crt - FAELGLAIFR | 72.6 | 10.0 | 29.3 | 12.0 |
| 503.2497 | 805.4124 | 10 | Ccar_Hbd - AEDIDLAMK | 67.8 | 10.0 | 27.0 | 12.0 |
| 666.3745 | 816.4825 | 10 | Ccar_Thl - DVTTVDLGATVIK | 79.7 | 10.0 | 32.8 | 12.0 |
| 464.2233 | 642.3246 | 10 | Ccar_Bcd - GMPGFSFGK | 65.0 | 10.0 | 25.6 | 12.0 |
| 595.3324 | 732.4403 | 10 | Ccar_EtfB - GTGEIVNKPFK | 74.5 | 10.0 | 30.3 | 12.0 |
| 679.8673 | 704.3686 | 10 | Ccar_EtfA - ELADLLGGTVAGSR | 80.7 | 10.0 | 33.3 | 12.0 |
| 502.7560 | 613.3668 | 10 | Clju_AcsB - DFEPVLER | 67.8 | 10.0 | 27.0 | 12.0 |
| 404.2398 | 496.2514 | 10 | Clju_Fhs - IVVGYTR | 60.6 | 10.0 | 23.4 | 12.0 |
| 351.2132 | 517.2980 | 10 | Clju_MeTr - AIEAGLK | 56.7 | 10.0 | 21.5 | 12.0 |

**Only Q1 and Q3 masses of fragments, which were used for quantification are shown. dwell time, declustering potential (DP), collision energy (CE), collision exit potential (CXP)**

# Supplementary results

## Product and biomass formation of *C. lju* pIM Hex#15 on different gas compositions

**Figure S 3 Maximum product concentrations and optical densities achieved by *C. lju* pIM Hex#15 after 382 h in standing serum bottle cultivations with different gas compositions as sole carbon and energy source.** Gas compositions used were: 33.3 % CO (purity ≥ 99 %), 33.3 % CO_2_ (purity ≥ 99.9 %) and 33.3 % H_2_ (purity ≥ 99.999%) or 20% CO_2_ (purity ≥ 99.9 %) and 80% H_2_ (purity ≥ 99.999 %). Data are means ± SD of n = 2 biological replicates.

## Genomic integration

The genomic integration locus of the Hex#15 construct in the *C. ljungdahlii* wild-type genome was determined by inverse PCR and proven by PCR analysis with primers spanning the integration locus and the borders of the construct with the genome (Figure S 4, Table S 8).

**
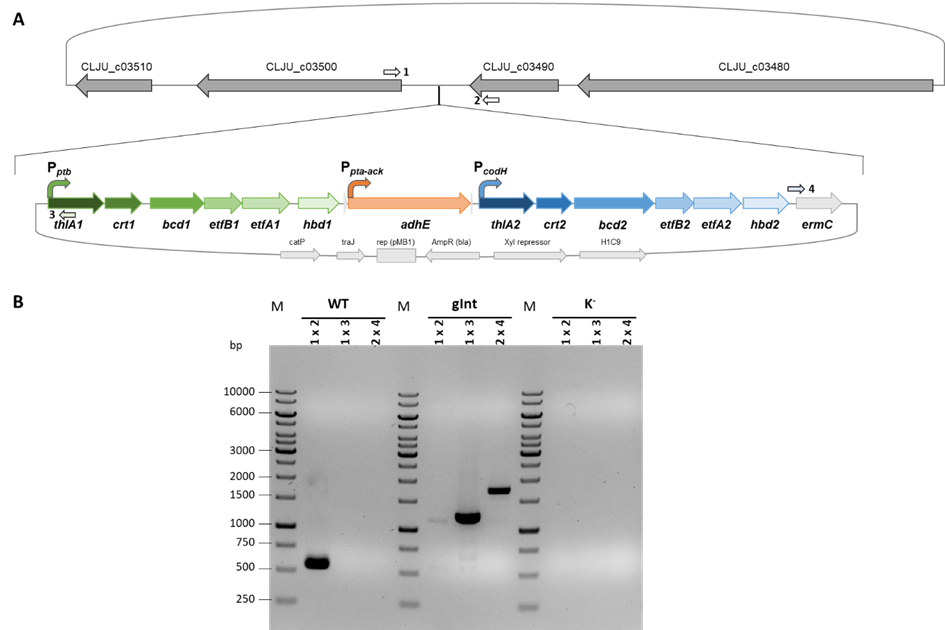
**

Figure S 4: Schematic overview of the genomic integration locus of the butanol/hexanol construct in *C. ljungdahlii* wildtype genome (A) and verification of the correct predicted position with three different control PCRs (B): GeneRuler 1 kb DNA-Ladder (M) (Thermo Fisher Scientific, Waltham, MA, USA); (1 x 2) primers spanning the integration locus resulting in a 556 bp band in *C. ljungdahlii* wildtype (WT) and a theoretical 18505 bp (not detected) band in *C. lju* Hex#15^gInt^ (gInt); (1 x 3) primers spanning the left border of the construct resulting in no band in WT and a 1210 bp band in gInt and (2 x 4) primers spanning the right border of the construct resulting in no band for WT and a 1750 bp band for gInt. Negative controls (K-) were performed without template resulting in no PCR products.

Table S 8: Oligo nucleotide primers for the verification of Hex#15 integration locus.

| # | Primer Name | Primer sequence 5’ → 3’ |
| --- | --- | --- |
| 1 | Clju_c03500_rev | CTTCAAGTTCCACTTCTATGCCTTCTG |
| 2 | Clju_c03490_for | TCAGACATGGATATCAGAAGAAGATGG |
| 3 | Hx_197 | TTATCTTTCAACTACTACAGCGGTTCCTTGTCCTCC |
| 4 | mlsR_for | GATAATATCTTTGAAATCGGCTCAGG |

## Excision of *ermC* using CRISPR/Cas9

The antibiotic resistance gene *ermC* was excised using CRISPR/Cas9, which was checked by PCR spanning the resistance gene (resulting in a 108 bp fragment in case the resistance gene was excised). Additionally, PCRs on the CRISPR plasmid and the Hex#15 construct were performed as control (Figure S 5, Table S 9). The successful removal of the *ermC* gene and the loss of the CRISPR plasmid were proven by PCR analysis and additionally by whole genome sequencing.


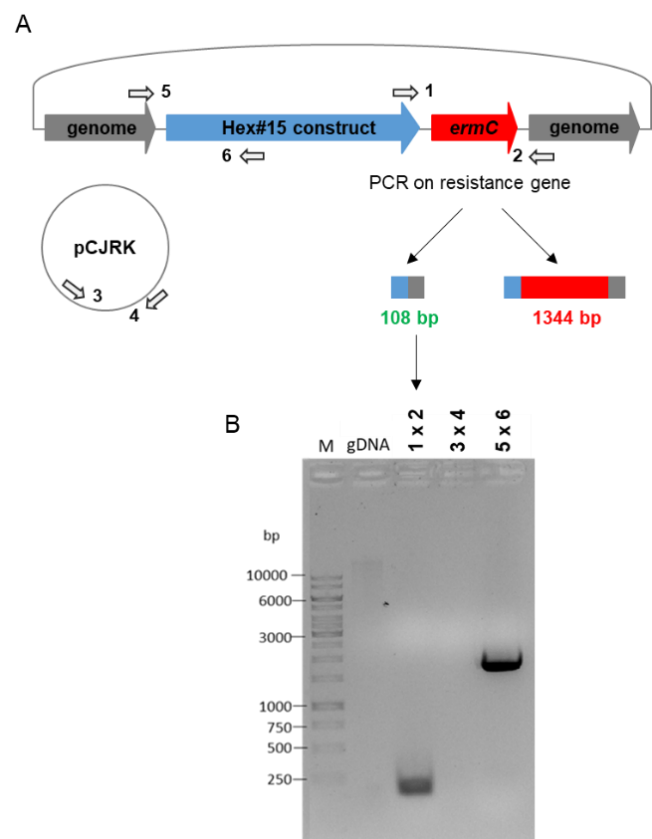


Figure S 5: A) Visualization of PCR to check for the removal of the antibiotic resistance gene *ermC.* PCR will result either in a 1344 bp product, if *ermC* is still present or a small 108 bp product, if *ermC* was removed successfully. B) GeneRuler 1 kb DNA-Ladder (M); genomic DNA of a single colony (gDNA); PCR analysis of a single colony (*C. lju* Hex#15gInt Δ*ermC* K1.4.2) to check for *ermC* loss (1 x 2, 108 bp/1344 bp), presence of CRISPR plasmid (3 x 4, 0 bp/1990 bp) and presence of a segment of the butanol/hexanol construct (5 x 6, 1750 bp).

Table S 9: Oligo nucleotide primers for the verification of *ermC* excision and loss of CRISPR plasmid.

| # | Primer Name | Primer sequence 5’ → 3’ |
| --- | --- | --- |
| 1 | mlsR_loss_for01 | AGGCCGGCGCGGCCGATTTAAATCCTC |
| 2 | mlsR_loss_rev01 | TTGGCTGATAAGTCCCCGGTCTACATTC |
| 3 | Gn034 | CCAAGGAGCTTTTTAAAGC |
| 4 | bgaR_for | TAGGATTTAGTGGTTCAAATGCTAATG |
| 5 | Clju_c03500_rev | CTTCAAGTTCCACTTCTATGCCTTCTG |
| 6 | CKLU_ThlaA1 rev | TTATCTTTCAACTACTACAGCGGTTCCTTGTCCTCC |

## Product formation of *C. ljungdahlii* with pIM Ccar1 and pIM Ccar1 on YTF medium

**Figure S 6 Product concentrations of cultures grown from single colonies of *C. ljungdahlii* wildtype after conjugation with plasmids for butanol/hexanol formation from *C. carboxidivorans*.** Cells were cultivated for one week in YTF medium supplemented with 200 µg L^-1^ D‑cycloserine, 4 µg L^-1^ clarithromycin and 4 µg L^-1^ thiamphenicol.

## Targeted proteomics data of *C. lju* pIM Hex#15 and *C. lju* Hex#15^gInt^

Table S 10: Enzyme content [ng_enzyme_ µg^-1^_soluble protein_] of butanol/hexanol pathway and native *C. ljungdahlii* enzymes in *C. lju* pIM Hex#15 and *C. lju* Hex#15^gInt^ in the early logarithmic (OD_600_ 0.3 – 0.5), late logarithmic (OD_600_ 0.5 – 1.0) and stationary growth phase (OD_600_ >1.0) in a 2-L fermentation with continuous gas supply with 20% CO_2_, 80% H_2_.

|  | enzyme content [ng_enzyme_ µg^-1^_soluble protein_] | | | | | | | |  |  |
| --- | --- | --- | --- | --- | --- | --- | --- | --- | --- | --- |
| Strain | | *C. lju* pIM Hex#15 | | | *C. lju* Hex#15^gInt^ | | | | |  |
| Growth phase | | early log | late log | stationary | | early log | late log | stationary | | |
|  | |  |  |  | |  |  |  | | |
| ThlA1 | | 0.16 | 0.14 | 0.14 | | 0.28 | 0.54 | 0.85 | | |
| Crt1 | | 0.33 | 0.22 | 0.20 | | 0.45 | 0.50 | 1.00 | | |
| Bcd1 | | 1.84 | 0.67 | 0.55 | | 3.11 | 2.65 | 2.66 | | |
| EtfB1 | | 0.98 | 0.52 | 0.40 | | 2.29 | 3.07 | 3.08 | | |
| EtfA1 | | 3.88 | 1.38 | 0.96 | | 7.75 | 9.76 | 8.30 | | |
| Hbd1 | | 2.67 | 1.88 | 1.49 | | 2.02 | 2.77 | 4.35 | | |
| ThlA2 | | 18.11 | 11.69 | 9.19 | | 18.56 | 18.32 | 22.46 | | |
| Crt2 | | 0.43 | 0.39 | 0.40 | | 0.43 | 0.50 | 0.68 | | |
| Bcd2 | | 8.39 | 8.68 | 7.40 | | 6.89 | 8.60 | 16.59 | | |
| EtfB2 | | 1.76 | 1.68 | 1.28 | | 1.70 | 1.64 | 1.55 | | |
| EtfA2 | | 1.76 | 2.37 | 2.50 | | 1.77 | 2.75 | 1.18 | | |
| Hbd2 | | 16.53 | 10.89 | 9.73 | | 14.30 | 13.13 | 9.92 | | |
| AdhE2 | | 0.06 | 2.25 | 0.75 | | 10.00 | 18.73 | 31.64 | | |
| AcsB | | 33.07 | 45.30 | 44.82 | | 39.39 | 49.81 | 68.73 | | |
| Fhs | | 18.35 | 24.39 | 28.80 | | 16.41 | 15.36 | 24.42 | | |
| MeTr | | 8.93 | 10.53 | 10.85 | | 9.63 | 9.92 | 12.32 | | |
| CatP | | 0.35 | 0.62 | 0.68 | | n.d. | n.d. | n.d. | | |

## Targeted proteomics data of *C. lju* Hex#15^gInt^ Ccar1^gInt^ cultivated with different pH profiles

Table S 11: Enzyme content [ng_enzyme_ µg^-1^_soluble protein_] of butanol/hexanol pathway and native *C. ljungdahlii* enzymes in *C. lju* Hex#15^gInt^ Ccar1^gInt^ in the early logarithmic (OD_600_ 0.3 – 0.5) and late logarithmic (OD_600_ 0.5 – 1.0) growth phase in 2 L fermentations with constant pH 5.9 (I) and unregulated pH (II) with continuous gas supply with 20% CO_2_, 80% H_2_.

| enzyme content [ng_enzyme_ µg^-1^_soluble protein_] | | | | |
| --- | --- | --- | --- | --- |
| Strain | *C. lju* Hex#15^gInt^ Ccar1^gInt^ | | | |
| fermentation | I | | II | |
| Growth phase | early log | late log | early log | late log |
|  |  |  |  |  |
| ThlA1 | 0.80 | 0.87 | 0.86 | 0.46 |
| Crt1 | 0.36 | 0.30 | 0.97 | 0.61 |
| Bcd1 | 4.98 | 6.35 | 11.66 | 7.98 |
| EtfB1 | 1.09 | 1.72 | 3.39 | 2.73 |
| EtfA1 | 5.73 | 8.70 | 19.67 | 12.34 |
| Hbd1 | 1.10 | 2.39 | 4.98 | 2.81 |
| ThlA2 | 21.43 | 23.16 | 32.05 | 25.23 |
| Crt2 | 0.47 | 0.65 | 1.45 | 0.92 |
| Bcd2 | 9.10 | 9.65 | 14.62 | 18.18 |
| EtfB2 | 1.77 | 1.92 | 4.22 | 2.73 |
| EtfA2 | 1.68 | 2.51 | 6.99 | 2.34 |
| Hbd2 | 8.90 | 8.18 | 15.35 | 11.71 |
| AdhE2 | 4.49 | 13.30 | 23.53 | 13.04 |
| Ccar_Crt | 0.22 | 0.32 | 0.14 | 0.16 |
| Ccar_Hbd | 3.37 | 2.70 | 2.74 | 1.96 |
| Ccar_Thl | 18.23 | 15.95 | 16.03 | 14.24 |
| Ccar_Bcd | 0.32 | 0.27 | 0.40 | 0.24 |
| Ccar_EtfB | 1.67 | 2.36 | 3.64 | 1.81 |
| Ccar_EtfA | 13.56 | 19.47 | 19.41 | 12.88 |
| AcsB | 41.64 | 45.01 | 76.65 | 68.80 |
| Fhs | 11.44 | 19.28 | 21.10 | 21.57 |
| MeTr | 14.28 | 14.81 | 25.10 | 23.50 |

**Supplementary References**

**Bengelsdorf, F. R., Poehlein, A., Linder, S., Erz, C., Hummel, T., Hoffmeister, S., Daniel, R. & Dürre, P.** (2016). Industrial Acetogenic Biocatalysts: A Comparative Metabolic and Genomic Analysis. *Front Microbiol* **7**, 1036.

**Gaida, S. M., Liedtke, A., Jentges, A. H., Engels, B. & Jennewein, S.** (2016). Metabolic engineering of *Clostridium cellulolyticum* for the production of n-butanol from crystalline cellulose. *Microb Cell Fact* **15**, 6.

**Gibson, D. G., Young, L., Chuang, R. Y., Venter, J. C., Hutchison, C. A., 3rd & Smith, H. O.** (2009). Enzymatic assembly of DNA molecules up to several hundred kilobases. *Nat Methods* **6**, 343-345.

**Lampe, D. J., Akerley, B. J., Rubin, E. J., Mekalanos, J. J. & Robertson, H. M.** (1999). Hyperactive transposase mutants of the Himar1 mariner transposon. *Proc Natl Acad Sci U S A* **96**, 11428-33.

**Naito, Y., Hino, K., Bono, H. & Ui-Tei, K.** (2015). CRISPRdirect: software for designing CRISPR/Cas guide RNA with reduced off-target sites. *Bioinformatics* **31**, 1120-1123.

**Philipps, G., de Vries, S. & Jennewein, S.** (2019). Development of a metabolic pathway transfer and genomic integration system for the syngas-fermenting bacterium Clostridium ljungdahlii. *Biotechnol Biofuels* **12**, 112.

**Phillips, J. R., Atiyeh, H. K., Tanner, R. S., Torres, J. R., Saxena, J., Wilkins, M. R. & Huhnke, R. L.** (2015). Butanol and hexanol production in *Clostridium carboxidivorans* syngas fermentation: Medium development and culture techniques. *Bioresour Technol* **190**, 114-121.

**Shevchuk, N. A., Bryksin, A. V., Nusinovich, Y. A., Cabello, F. C., Sutherland, M. & Ladisch, S.** (2004). Construction of long DNA molecules using long PCR-based fusion of several fragments simultaneously. *Nucleic Acids Res* **32**, e19.
